# Fault-tolerance in metric dimension of boron nanotubes lattices

**DOI:** 10.3389/fncom.2022.1023585

**Published:** 2023-01-25

**Authors:** Zafar Hussain, Muhammad Mobeen Munir

**Affiliations:** ^1^Department of Mathematics and Statistics, The University of Lahore, Lahore, Pakistan; ^2^Department of Mathematics, University of the Punjab, Lahore, Pakistan

**Keywords:** metric dimension, fault-tolerant metric dimension, boron tubes, metric basis, 2-d lattices, resolving set

## Abstract

The concept of resolving set and metric basis has been very successful because of multi-purpose applications both in computer and mathematical sciences. A system in which failure of any single unit, another chain of units not containing the faulty unit can replace the originally used chain is called a fault-tolerant self-stable system. Recent research studies reveal that the problem of finding metric dimension is NP-hard for general graphs and the problem of computing the exact values of fault-tolerant metric dimension seems to be even harder although some bounds can be computed rather easily. In this article, we compute closed formulas for the fault-tolerant metric dimension of lattices of two types of boron nanotubes, namely triangular and alpha boron. These lattices are formed by cutting the tubes vertically. We conclude that both tubes have constant fault tolerance metric dimension 4.

## 1. Introduction

Computer networks are graphs with vertices representing hosts, servers, or hubs, and edges as connecting mediums between them. Vertex is actually a possible location to find faults or some damaged devices in a computer network. This idea somehow created an urge in Slater and independently in Harary and Melter (1976) to uniquely recognize each vertex of a graph in a network so that fault could be controlled efficiently. Thus, the basis for the notion of locating sets and locating the number of graphs came into existence. Since then, the resolving sets have been extensively investigated (Buczkowski et al., 2003; Caceres et al., 2005; Javaid et al., 2008). The resolving set contributes to various areas such as network discovery (Khuller et al., 1994, 1996), connected joins in graphs, strategies for the mastermind games (Chvatal, 1983), applications of pattern recognition, combinatorial optimization, image processing (Melter and Tomescu, 1984), pharmaceutical chemistry, and game theory.

A moving point in a graph may be located by finding the distance from the point to the collection of sonar stations that have been properly positioned in the graph. Thus finding a minimal but sufficiently large set of labeled vertices such that a robot can find its position, is a well-established problem known as robot navigation. This sufficiently large set of labeled vertices is a resolving set of the graph space and the corresponding cardinality is the metric dimension. Similarly, on another node, a real-world problem is the study of networks whose structure has not been imposed by a central authority but has arisen from local and distributed processes. It is very difficult and expensive to obtain a map of all nodes and the links between them. A commonly used technique is to obtain a local view of the network from various locations and combine them to obtain a good approximation for the real network. Metric dimension also has some applications in this aspect as well.

Consider a simple, connected graph *G*, and metric *d*_*G*_ : *V*(*G*) × *V*(*G*) → ℕ ∪ {0}, where ℕ is the set of positive integers and *d*_*G*_(*x, y*) is the minimum number of edges in any path between x and y. Let *W* = {*w*_1_, *w*_2_, ..., *w*_*k*_} be an ordered set of vertices of *G* and let *v* be a vertex of *G*. The representation *r*(*v*|*W*) of *v* with respect to *W* is the *k*−tuple (*d*_*G*_(*v, w*_1_), *d*_*G*_(*v, w*_2_), ..., *d*_*G*_(*v, w*_*k*_)). If distinct vertices of *G* have a distinct representation with respect to *W*, then *W* is called a resolving set of *G* (see Khuller et al., 1994, 1996; Buczkowski et al., 2003; Javaid et al., 2008). Such a resolving set with minimum cardinality is a basis of *G* and the metric dimension of *G*, denoted by β(*G*) is its cardinality.

Buczkowski et al. (2003) established the metric dimension of wheel *W*_*n*_ to be $\left\lfloor \frac{2n+2}{5}\right\rfloor$ for *n* ≥ 7. Caceres et al. (2005) found that the metric dimension of fan to be $\left\lfloor \frac{2n+2}{5}\right\rfloor$ for *n* ≥ 7. Tomescu and Javaid (2007) determined the dimension of Jahangir graphs *J*_2*n*_ to be $\left\lfloor \frac{2n}{3}\right\rfloor$ for all *n* ≥ 4. A particular metric-feature of the family of graphs is independent of metric dimension on the particular element of the family. A connected graph has a constant metric dimension if β(*G*) = *k*, where *k* ∈ ℕ is fixed. This feature has been presented in Imran et al. (2012) study. Imran et al. (2013) computed metric dimension of flower graph and some families of convex polytopes. Chartrand et al. (2000) proved that a graph has a constant metric dimension of 1 if it is a path. Ali et al. (2012) computed partial results of the metric dimension of the Mobius ladder, whereas Munir et al. (2017) computed exact and complete results for the metric dimension of the Mobius Ladders. Hussain et al. (2010) computed upper bounds for the metric dimension and partition dimension of generalized Mobius ladders. Poisson and Zhang (2002) computed the metric dimension of uni-cyclic graphs. For detailed review of some variants of dimensions, please see Tomescu and Imran (2009), Manuel (2010), Afzal and Imran (2014), Raza et al. (2018).

Recent development in this context has paved way for a new related concept known as fault-tolerance in the metric dimension. Suppose that, in a network, *n* processing units are interlinked, and of these units, forming a chain of maximal length are used to solve some task. To have a fault-tolerant self-stable system, it is necessary that in the case of failure of any single unit, another chain of units not containing the faulty unit can replace the originally used chain. Thus, a fault-tolerant design enables a system to continue its intended operation, possibly at a reduced level, rather than failing completely. The units and the links are represented by graphs refer to Slater (1975, 1998, 2002) and Hayes (1985). A resolving set Ś is considered fault-tolerant if Ś\{*v*} is also a resolving set, for each *v* ∈ Ś, and the fault-tolerant metric dimension, β′(*G*), is the minimum cardinality of such Ś. A family G of connected graphs is said to have a constant fault-tolerant metric dimension if it is independent of any choice of a member of that family. Fault-tolerant designs are widely used in engineering and computer sciences (Hayes, 1985). Slater (2002) introduced the study of fault-tolerant locating-dominating sets. Hernando et al. (2008) introduced the idea of a single fault-tolerant metric dimension. The authors discussed the single fault-tolerant metric dimension of trees. They also proved that fault-tolerant metric dimension is bounded by a function of the metric dimension irrespective of the choice of the graph given be $\overset{´}{\beta \left(G\right)}\leq \beta \left(G\right)\left(1+2.5^{\beta \left(G\right)-1}\right)$. Javed et al. discussed fault tolerance in resolvability (Chaudhry et al., 2010) and computed fault tolerant metric dimension of some graphs (Javaid et al., 2009). It is easy to gather that $\overset{´}{\beta \left(G\right)}\geq \beta \left(G\right)+1$ (Javaid et al., 2009). Shabbir and Zumfererscu (2016) discussed fault tolerance in triangular lattice networks.

**Figure 1:**
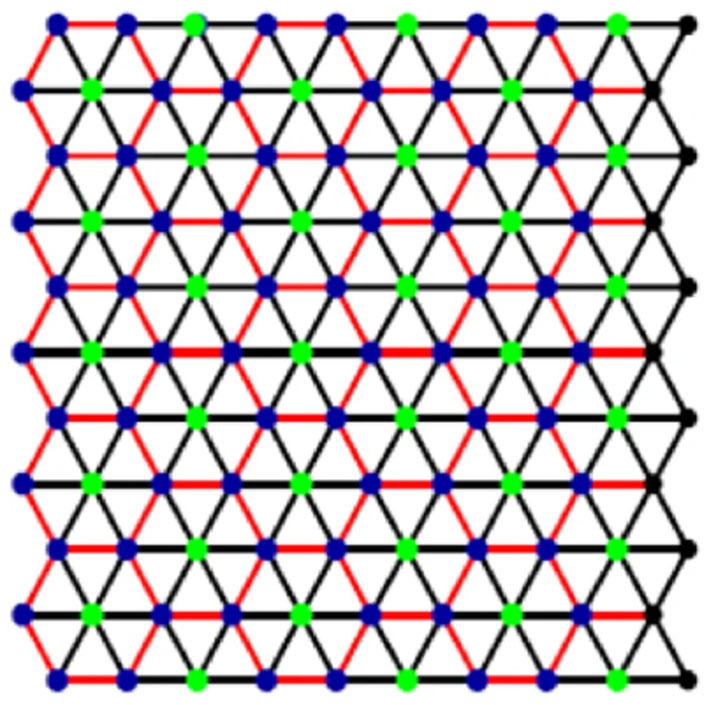
Triangular boron nanotube lattice, *T*_*k*×*l*_.

The subject matter of this article is fault tolerance in metric dimension of lattices of two types of boron nanotubes, alpha boron α_*m*×*n*_ and triangular boron nanotubes *T*_*m*×*n*_. These lattices are formed by cutting both tubes vertically. Kwun et al. (2018) computed *M*-polynomials and related topological indices of these tubes and drew some nice comparative remarks about these tubes. Recently, Hussain et al. (2018) computed the metric dimension of α_*m*×*n*_. Figure 1 presents a triangular boron tube lattice.

**Figure 2:**
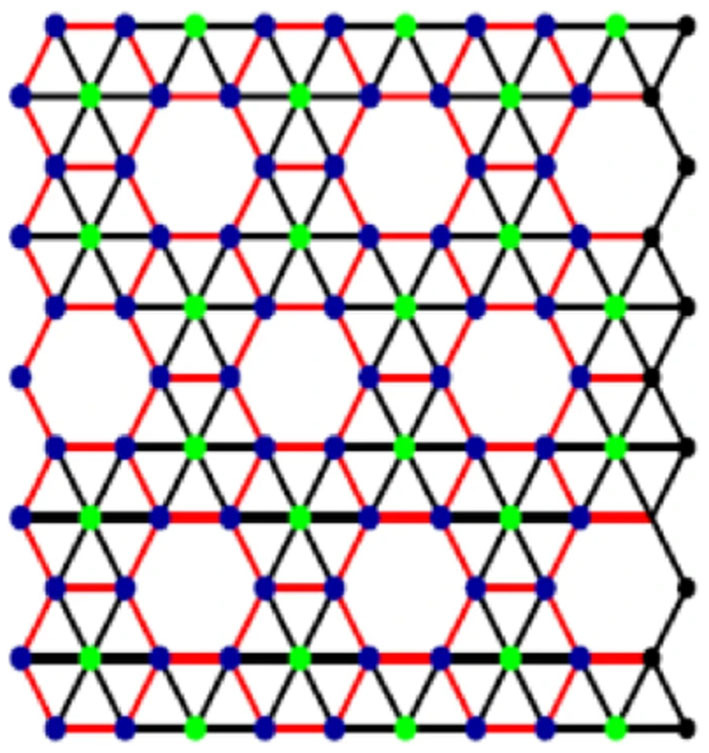
Alpha boron nanotube lattice, α_*k*×*l*_.

Figure 2 presents alpha boron tube lattice.

## 2. Main results

This section discusses main results. At first, we compute the fault-tolerant metric dimension of triangular boron tubes and then we focus on alpha boron tubes.

**Theorem 2.1**. Let *T*_*k,l*_ denote the graph of *k* × *l*, 2D-lattice of triangular boron nano tubes. Then $\beta^{\prime }\left(T_{k,l}\right)\leq 4$.

**Figure 3:**
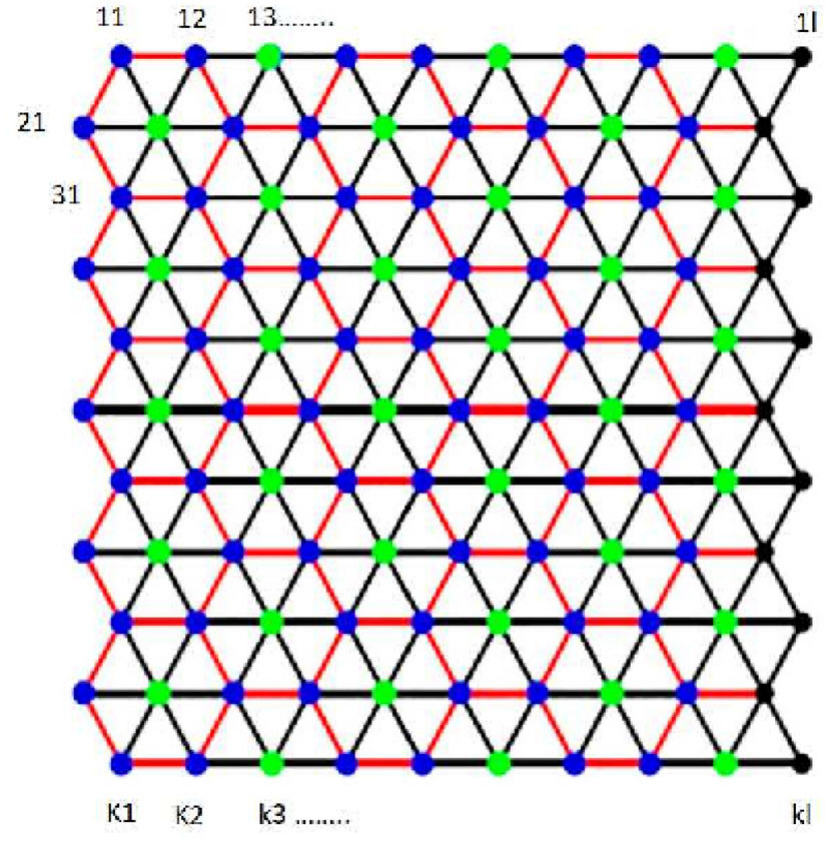
Labeling of vertices of *T*_*k,l*_.

*Proof*. The vertex set of *T*_*k,l*_ is {*v*_1,1_, *v*_1,2_, *v*_1,3_, ...., *v*_1,*l*_, *v*_2,1_, *v*_2,2_, *v*_2,3_, ...., *v*_2,*l*_, *v*_3,1_, *v*_3,2_, *v*_3,3_, ......, *v*_3,*l*_, ......, *v*_*k*,1_, *v*_*k*,2_, *v*_*k*,3_, ...., *v*_*k,l*_}. Let *F* = {*v*_1,1_, *v*_1,*l*_, *v*_*k*,1_, and*v*_*k,l*_}. We prove that *F* is an FTRS for *T*_*k,l*_. In general, the tube is “cut” vertically, and the upper left triangle is pointing up as shown in Figure 3. This figure presents labeling of vertices that becomes optimal for the basis of fault tolerance.

**Case I**: Let *k* ≤ 2*l*, *k* is odd.

We give the distance vectors of all vertices *v*_*a,b*_ of *T*_*k,l*_ relative to *F*.

For *a* = 1


$$\begin{array}{l} \;r\left(v_{a,b}|F\right)\\ =\{\begin{array}{c} \left(b-1,l-b,k-1,\frac{k-1}{2}+l-b\right),\text{if }1\leq b\leq l-\frac{k+1}{2}\\ \left(b-1,l-b,k-1,k-1\right),\text{if }l-\frac{k-1}{2}\leq b\leq \frac{k+1}{2}\\ \left(b-1,l-b,\frac{k-3}{2}+b,k-1\right),\text{if }\frac{k+3}{2}\leq b\leq l \end{array} \end{array}$$

For *a* = 2


$$\begin{array}{l} \;r\left(v_{a,b}|F\right)\\ =\{\begin{array}{c} \left(1,l,k-2,\frac{k-1}{2}+l-b\right),\text{if }b=1\\ \left(b-1,1+l-b,k-2,\frac{k-1}{2}+l-b\right),\text{if }2\leq b\leq l-\frac{k-1}{2}\\ \left(b-1,1+l-b,k-2,k-2\right),\text{if }l-\frac{k-3}{2}\leq b\leq \frac{k+1}{2}\\ \left(b-1,1+l-b,\frac{k-5}{2}+b,k-2\right),\text{if }\frac{k+3}{2}\leq b\leq l \end{array} \end{array}$$

In general, if *k* is odd, *x* = *k* − *a*, *a* is odd, and $3\leq a\leq \left\lceil \frac{k}{2}\right\rceil$ and then


$$\begin{array}{l} \;r\left(v_{a,b}|F\right)\\ =\{\begin{array}{c} \left(a-1,\left\lfloor \frac{a}{2}\right\rfloor +l-b,x,n+\frac{x}{2}-b\right),\text{if }1\leq b\leq \frac{a-1}{2}\\ \left(\left\lfloor \frac{a-3}{2}\right\rfloor +b,\left\lfloor \frac{a}{2}\right\rfloor +l-b,x,\frac{x}{2}+l-b\right),\text{if }\frac{a+1}{2}\\ \leq b\leq \frac{x+2}{2}\\ \left(\left\lfloor \frac{a-3}{2}\right\rfloor +b,\left\lfloor \frac{a}{2}\right\rfloor +l-b,\frac{x-2}{2}+b,\frac{x}{2}+l-b\right),\\ \text{if }\frac{x+4}{2}\leq b\leq l-\frac{x+2}{2}\\ \left(\left\lfloor \frac{a-3}{2}\right\rfloor +b,\left\lfloor \frac{a}{2}\right\rfloor +l-b,\frac{x-2}{2}+b,x\right),\text{if }l-\frac{x}{2}\\ \leq b\leq l-\frac{a-1}{2}\\ \left(\left\lfloor \frac{a-3}{2}\right\rfloor +b,a-1,\frac{x-2}{2}+b,x\right),\text{if }l-\frac{a-3}{2}\leq b\leq l \end{array} \end{array}$$

If *k* is odd, *a* is odd, and $\left\lceil \frac{k}{2}\right\rceil +1\leq a\leq k$ then


$$\begin{array}{l} \;r\left(v_{a,b}|F\right)\\ =\{\begin{array}{c} \left(a-1,\left\lfloor \frac{a}{2}\right\rfloor +l-b,x,\frac{x}{2}+l-b\right),\text{if }1\leq b\leq \frac{x+2)}{2}\\ \left(a-1,\left\lfloor \frac{a}{2}\right\rfloor +l-b,\frac{x-2}{2}+b,\frac{x}{2}+l-b\right),\text{if }\frac{x+4}{2}\\ \leq b\leq \frac{a+1}{2}\\ \left(\left\lfloor \frac{a-3}{2}\right\rfloor +b,\left\lfloor \frac{a}{2}\right\rfloor +l-b,\frac{x-2}{2}+b,\frac{x}{2}+l-b\right),\text{if }\frac{a+3}{2}\\ \leq b\leq l-\frac{a+1}{2}\\ \left(\left\lfloor \frac{a-3}{2}\right\rfloor +b,a-1,\frac{x-2}{2}+b,\frac{x}{2}+l-b\right),\text{if }l-\frac{a-1}{2}\\ \leq b\leq l-\frac{x+2}{2}\\ \left(\left\lfloor \frac{a-3}{2}\right\rfloor +b,a-1,\frac{x-2}{2}+b,x\right),\text{if }l-\frac{x}{2}\leq b\leq l \end{array} \end{array}$$

If *k* is odd, *a* is even, and $3<a\leq \left\lceil \frac{k}{2}\right\rceil$ then


$$\begin{array}{l} \;r\left(v_{a,b}|F\right)\\ =\{\begin{array}{c} \left(a-1,\left\lfloor \frac{a}{2}\right\rfloor +l-b,x,\frac{x+1}{2}+l-b\right),\text{if }1\leq b\leq \frac{a}{2}\\ \left(\left\lfloor \frac{a-3}{2}\right\rfloor +b,\left\lfloor \frac{a}{2}\right\rfloor +l-b,x,\frac{x+1}{2}+l-b\right),\text{if }\frac{a+2}{2}\\ \leq b\leq \frac{x+3}{2}\\ \left(\left\lfloor \frac{a-3}{2}\right\rfloor +b,\left\lfloor \frac{a}{2}\right\rfloor +l-b,x,x\right),\text{if }\frac{x+5}{2}\\ \leq b\leq l-\frac{k-\left(a-1\right)}{2}\\ \left(\left\lfloor \frac{a-3}{2}\right\rfloor +b,\left\lfloor \frac{a}{2}\right\rfloor +l-b,\frac{x-3}{2}+b,x\right),\text{if }l-\frac{x-1}{2}\\ \leq b\leq l-\frac{a-2}{2}\\ \left(\left\lfloor \frac{a-3}{2}\right\rfloor +b,a-1,\frac{x-3}{2}+b,x\right),\text{if }l-\frac{a-4}{2}\leq b\leq l \end{array} \end{array}$$

If *k* is odd, *a* is even, and $\left\lceil \frac{k}{2}\right\rceil +1\leq a\leq k$ then


$$\begin{array}{l} \;r\left(v_{a,b}|F\right)\\ =\{\begin{array}{c} \left(a-1,l+\left\lfloor \frac{a}{2}\right\rfloor -b,x,\frac{x+1}{2}+l-b\right),\text{if }1\leq b\leq \frac{x+1}{2}\\ \left(a-1,l+\left\lfloor \frac{a}{2}\right\rfloor -b,\frac{x-3}{2}+b,\frac{x+1}{2}+l-b\right),\text{if }\frac{x+3}{2}\\ \leq b\leq \frac{a}{2}\\ \left(\left\lfloor \frac{a-3}{2}\right\rfloor +b,l+\left\lfloor \frac{a}{2}\right\rfloor -b,\frac{x-3}{2}+b,\frac{x+1}{2}+l-b\right),\text{if }\frac{a+2}{2}\\ \leq b\leq l-\frac{a}{2}\\ \left(\left\lfloor \frac{a-3}{2}\right\rfloor +b,a-1,\frac{x-3}{2}+b,\frac{x+1}{2}+l-b\right),\text{if }l-\frac{a-2}{2}\\ \leq b\leq l-\frac{x+1}{2}\\ \left(\left\lfloor \frac{a-3}{2}\right\rfloor +b,a-1,\frac{x-3}{2}+b,x\right),\text{if }l-\frac{x-1}{2}\leq b\leq l \end{array} \end{array}$$

For *a* = 2*l* − 1, *r*(*V*_*a,b*_|*S*) = (2*l* − 2, 2*l* − 2, *q* − 1, *l* − *q*), if 1 ≤ *q* ≤ *l*

If *k* is even, *a* is odd, and $3\leq a\leq \left\lceil \frac{k}{2}\right\rceil$ then


$$\begin{array}{l} \;r\left(v_{a,b}|F\right)\\ =\{\begin{array}{c} \left(a-1,l+\left\lfloor \frac{a}{2}\right\rfloor -b,x,\frac{x-1}{2}+l-b\right),\text{if }1\leq b\leq \frac{a-1}{2}\\ \left(\left\lfloor \frac{a-3}{2}\right\rfloor +b,\left\lfloor \frac{a}{2}\right\rfloor +l-b,x,\frac{x-1}{2}+l-b\right),\text{if }\frac{a+1}{2}\\ \leq b\leq l-\frac{x+3}{2}\\ \left(\left\lfloor \frac{a-3}{2}\right\rfloor +b,\left\lfloor \frac{a}{2}\right\rfloor +l-b,x,x\right),\text{if }l-\frac{x+1}{2}\\ \leq b\leq \frac{x+1}{2}\\ \left(\left\lfloor \frac{a-3}{2}\right\rfloor +b,\left\lfloor \frac{a}{2}\right\rfloor +l-b,\frac{x-1}{2}+b,x\right),\text{if }\frac{x+3}{2}\\ \leq b\leq l-\frac{a+1}{2}\\ \left(\left\lfloor \frac{a-3}{2}\right\rfloor +b,a-1,\frac{x-1}{2}+b,x\right),\text{if }l-\frac{a-1}{2}\leq b\leq l \end{array} \end{array}$$

If *a* is odd, *k* is even, and $\left\lceil \frac{k}{2}\right\rceil +1\leq a\leq k$ then


$$\begin{array}{l} \;r\left(v_{a,b}|F\right)\\ =\{\begin{array}{c} \left(a-1,\left\lfloor \frac{a}{2}\right\rfloor +l-b,x,\frac{x-1}{2}+l-b\right),\text{if }1\leq b\leq \frac{x+1}{2}\\ \left(a-1,\left\lfloor \frac{a}{2}\right\rfloor +l-b,\frac{x-1}{2}+b,\frac{x-1}{2}+l-b\right),\text{if }\frac{x+3}{2}\\ \leq b\leq l-\frac{a+1}{2}\\ \left(a-1,a-1,b+\frac{x-1}{2},\frac{x-1}{2}+l-b\right),\text{if }l-\frac{a-1}{2}\\ \leq b\leq \frac{a-1}{2}\\ \left(\left\lfloor \frac{a-3}{2}\right\rfloor +b,a-1,\frac{x-1}{2}+b,l+\frac{x-1}{2}-b\right),\text{if }\frac{a+1}{2}\\ \leq b\leq l-\frac{x+3}{2}\\ \left(\left\lfloor \frac{a-3}{2}\right\rfloor +b,a-1,\frac{x-1}{2}+b,x\right),\text{if }l-\frac{x+1}{2}\\ \leq b\leq l \end{array} \end{array}$$

If *k* is even, *a* is even, and $3<a\leq \left\lceil \frac{k}{2}\right\rceil$ then


$$\begin{array}{l} \;r\left(v_{a,b}|F\right)\\ =\{\begin{array}{c} \left(a-1,\left\lfloor \frac{a}{2}\right\rfloor +l-b,x,\frac{k-a}{2}+l-b\right),\text{if }1\leq b\leq \frac{a}{2}\\ \left(\left\lfloor \frac{a-3}{2}\right\rfloor +b,\left\lfloor \frac{a}{2}\right\rfloor +l-b,x,\frac{x}{2}+l-b\right),\text{if }\frac{a+2}{2}\\ \leq b\leq l-\frac{x+2}{2}\\ \left(\left\lfloor \frac{a-3}{2}\right\rfloor +b,\left\lfloor \frac{a}{2}\right\rfloor +l-b,\frac{x-2}{2}+b,\frac{x}{2}+l-b\right),\text{if }l-\frac{x}{2}\\ \leq b\leq \frac{k-\left(a-2\right)}{2}\\ \left(\left\lfloor \frac{a-3}{2}\right\rfloor +b,\left\lfloor \frac{a}{2}\right\rfloor +l-b,\frac{x-2}{2}+b,k-a\right),\text{if }\frac{x+4}{2}\\ \leq b\leq l-\frac{a-2}{2}\\ \left(\left\lfloor \frac{a-3}{2}\right\rfloor +b,a-1,\frac{x-2}{2}+b,x\right),\text{if }l-\frac{a-4}{2}\leq b\leq l \end{array} \end{array}$$

If *k* is even, *a* is even, and $\left\lceil \frac{k}{2}\right\rceil +1\leq a\leq k$ then


$$\begin{array}{l} \;r\left(v_{a,b}|F\right)\\ =\{\begin{array}{c} \left(a-1,\left\lfloor \frac{a}{2}\right\rfloor +l-b,x,\frac{x}{2}+l-b\right),\text{if }1\leq b\leq \frac{x+2}{2}\\ \left(a-1,\left\lfloor \frac{a}{2}\right\rfloor +l-b,\frac{x-2}{2}+b,\frac{x}{2}+l-b\right),\text{if }\frac{x+4}{2}\\ \leq b\leq l-\frac{a-2}{2}\\ \left(a-1,a-1,\frac{x-2}{2}+b,\frac{x}{2}+l-b\right),\text{if }l-\frac{a-4}{2}\leq b\leq \frac{a}{2}\\ \left(\left\lfloor \frac{a-3}{2}\right\rfloor +b,a-1,\frac{x-2}{2}+b,\frac{x}{2}+l-b\right),\text{if }\frac{a+2}{2}\\ \leq b\leq l-\frac{x+2}{2}\\ \left(\left\lfloor \frac{a-3}{2}\right\rfloor +b,a-1,\frac{x-2}{2}+b,x\right),\text{if }l-\frac{x}{2}\leq b\leq l \end{array} \end{array}$$

For *a* = 2*l*, *r*(*V*_*a,b*_|*S*) = (2*l* − 1, 2*l* − 1, *b* − 1, *l* − *b*), if 1 ≤ *b* ≤ *l*

For *a* = 2*l* − 1, *r*(*V*_*a,b*_|*S*) = (2*l* − 2, 2*l* − 2, *q* − 1, *l* − *q*), if 1 ≤ *q* ≤ *l*

If *k* is even, *a* is odd, and $3\leq a\leq \left\lceil \frac{k}{2}\right\rceil$ then


$$\begin{array}{l} \;r\left(V_{a,b}|F\right)\\ =\{\begin{array}{c} \left(a-1,\left\lfloor \frac{a}{2}\right\rfloor +l-b,x,\frac{x-1}{2}+l-b\right),\text{if }1\leq b\leq \frac{a-1}{2}\\ \left(\left\lfloor \frac{a-3}{2}\right\rfloor +b,\left\lfloor \frac{a}{2}\right\rfloor +l-b,x,\frac{x-1}{2}+l-b\right),\text{if }\frac{a+1}{2}\\ \leq b\leq l-\frac{x+3}{2}\\ \left(\left\lfloor \frac{a-3}{2}\right\rfloor +b,\left\lfloor \frac{a}{2}\right\rfloor +l-b,x,x\right),\text{if }l-\frac{x+1}{2}\leq b\leq \frac{x+1}{2}\\ \left(\left\lfloor \frac{a-3}{2}\right\rfloor +b,\left\lfloor \frac{a}{2}\right\rfloor +l-b,\frac{x-1)}{2}+b,k-a\right),\text{if }\frac{x+3}{2}\\ \leq b\leq l-\frac{a+1}{2}\\ \left(\left\lfloor \frac{a-3}{2}\right\rfloor +b,a-1,\frac{x+1}{2}+b,x\right),\text{if }l-\frac{a-1}{2}\leq b\leq l \end{array} \end{array}$$

If *k* is even, *a* is odd, and $\left\lceil \frac{k}{2}\right\rceil +1\leq a\leq k$ then


$$\begin{array}{l} \;r\left(V_{a,b}|F\right)\\ =\{\begin{array}{c} \left(a-1,\left\lfloor \frac{a}{2}\right\rfloor +l-b,x,l+\frac{x-1}{2}-b\right),\text{if }1\leq b\leq \frac{x+1}{2}\\ \left(a-1,\left\lfloor \frac{a}{2}\right\rfloor +l-b,\frac{x-1}{2}+b,l+\frac{x-1}{2}-b\right),\text{if }\frac{x+3}{2}\\ \leq b\leq l-\frac{a+1}{2}\\ \left(a-1,a-1,\frac{x-1}{2}+b,l+\frac{x-1}{2}-b\right),\text{if }l-\frac{a-1}{2}\\ \leq b\leq \frac{a-1}{2}\\ \left(\left\lfloor \frac{a-3}{2}\right\rfloor +b,a-1,\frac{x-1}{2}+b,l+\frac{x-1}{2}-b\right),\text{if }\frac{a+1}{2}\\ \leq b\leq l-\frac{x+3}{2}\\ \left(\left\lfloor \frac{a-3}{2}\right\rfloor +b,a-1,\frac{x-1}{2}+b,x\right),\text{if }l-\frac{x+1}{2}\leq b\leq l \end{array} \end{array}$$

If *k* is even, *a* is even, and $3<a\leq \left\lceil \frac{k}{2}\right\rceil$ then


$$\begin{array}{l} \;r\left(V_{a,b}|F\right)\\ =\{\begin{array}{c} \left(a-1,\left\lfloor \frac{a}{2}\right\rfloor +l-b,x,\frac{x}{2}+l-b\right),\text{if }1\leq b\leq \frac{a}{2}\\ \left(\left\lfloor \frac{a-3}{2}\right\rfloor +b,\left\lfloor \frac{a}{2}\right\rfloor +l-b,x,\frac{x}{2}+l-b\right),\text{if }\frac{a+2}{2}\\ \leq b\leq l-\frac{x+2}{2}\\ \left(\left\lfloor \frac{a-3}{2}\right\rfloor +b,\left\lfloor \frac{a}{2}\right\rfloor +l-b,\frac{x-2}{2}+b,\frac{x}{2}+l-b\right),\\ \text{if }l-\frac{x}{2}\leq b\leq \frac{x+2}{2}\\ \left(\left\lfloor \frac{a-3}{2}\right\rfloor +b,\left\lfloor \frac{a}{2}\right\rfloor +l-b,\frac{x-2}{2}+b,x\right),\text{if }\frac{x+4}{2}\\ \leq b\leq l-\frac{b-2}{2}\\ \left(\left\lfloor \frac{a-3}{2}\right\rfloor +b,a-1,\frac{x-2}{2}+b,x\right),\text{if }l-\frac{a-4}{2}\leq b\leq l \end{array} \end{array}$$

If *k* is even, *a* is even, and $\left\lceil \frac{k}{2}\right\rceil +1\leq a\leq k$ then


$$\begin{array}{l} \;r\left(V_{a,b}|F\right)\\ =\{\begin{array}{c} \left(a-1,\left\lfloor \frac{a}{2}\right\rfloor +l-b,x,\frac{x}{2}+l-b\right),\text{if }1\leq b\leq \frac{x+2}{2}\\ \left(a-1,\left\lfloor \frac{a}{2}\right\rfloor +l-b,\frac{x-2}{2}+b,\frac{x}{2}+l-b\right),\text{if }\frac{x+4}{2}\\ \leq b\leq l-\frac{a-2}{2}\\ \left(a-1,a-1,\frac{x-2}{2}+b,\frac{x}{2}+l-b\right),\text{if }l-\frac{a-4}{2}\leq b\leq \frac{a}{2}\\ \left(\left\lfloor \frac{a-3}{2}\right\rfloor +b,a-1,\frac{x-2}{2}+b,\frac{x}{2}+l-b\right),\\ \text{if }\frac{a+2}{2}\leq b\leq l-\frac{x+2}{2}\\ \left(\left\lfloor \frac{a-3}{2}\right\rfloor +b,a-1,\frac{x-2}{2}+b,x\right),\text{if }l-\frac{x}{2}\leq b\leq l \end{array} \end{array}$$

For *a* = 2*l*, *r*(*V*_*a,b*_|*S*) = (2*l* − 1, 2*l* − 1, *b* − 1, *l* − *b*), if 1 ≤ *b* ≤ *l*

**Case II**: Let *k* > 2*l*.

Let *y* = *rl* + *p* − 1. If *r* ≥ 2, *rl* < *k* ≤ (*r* + 1)*l*, and *k* is odd.

For *a* = *rl* + *p*, where 1 ≤ *p* ≤ *l* and *a* is odd


$$\begin{array}{l} \;r\left(V_{a,b}|F\right)\\ =\{\begin{array}{c} \left(y,y,x,l+\frac{k-\left(rl+p\right)}{2}-b\right),\text{if }1\leq b\leq \frac{k-\left(rl+p-2\right)}{2}\\ \left(y,y,\frac{k-\left(rl+p+2\right)}{2}+b,l+\frac{k-\left(rl+p\right)}{2}-b\right),\\ \text{if }\frac{k-\left(rl+p-4\right)}{2}\leq b\leq l-\frac{k-\left(rl+p-2\right)}{2}\\ \left(y,y,\frac{k-\left(rl+p+2\right)}{2}+b,k-a\right),\text{if }l-\frac{k-\left(rl+p\right)}{2}\leq b\leq l \end{array} \end{array}$$

If *a* is even


$$\begin{array}{l} \;r\left(V_{a,b}|F\right)\\ =\{\begin{array}{c} \left(y,y,x,l+\frac{k-\left(y\right)}{2}-b\right),\text{if }1\leq b\leq \frac{k-\left(rl+p-3\right)}{2}\\ \left(y,y,\frac{k-\left(rl+p+3\right)}{2}+b,l+\frac{k-\left(rl+p-1\right)}{2}-b\right),\\ \text{if }\frac{k-\left(rl+p-5\right)}{2}\leq b\leq l-\frac{p-\left(rl+p-1\right)}{2}\\ \left(y,y,\frac{k-\left(rl+p+3\right)}{2}+b,k-a\right),\text{if }l-\frac{k-\left(rl+p+1\right)}{2}\leq b\leq l \end{array} \end{array}$$

If *k* is even, *r* ≥ 2, and *rl* < *k* ≤ (*r* + 1)*l* then

Let *y* = *rl* + *p* − 1. For *a* = *rl* + *p* where 1 ≤ *p* ≤ *l* and *a* is odd


$$\begin{array}{l} \;r\left(V_{a,b}|F\right)\\ =\{\begin{array}{c} \left(y,y,m-a,n+\frac{k-\left(rl+p+1\right)}{2}-b\right),\text{if }1\leq b\leq \frac{k-y}{2}\\ \left(y,y,\frac{k-\left(rl+p+1\right)}{2}+b,l+\frac{k-\left(rl+p+1\right)}{2}-b\right),\\ \text{if }\frac{k-\left(rl+p-3\right)}{2}\leq b\leq l-\frac{k-\left(rl+p-3\right)}{2}\\ \left(y,y,\frac{k-\left(rl+p+1\right)}{2}+b,k-a\right),\text{if }l-\frac{k-y}{2}\leq b\leq l \end{array} \end{array}$$

If *a* is even


$$\begin{array}{l} \;r\left(V_{a,b}|F\right)\\ =\{\begin{array}{c} \left(y,y,x,\frac{k-\left(rl+p\right)}{2}-b\right)+l,\text{if }1\leq b\leq \frac{k-\left(rl+p-2\right)}{2}\\ \left(y,y,\frac{m-\left(rl+p+2\right)}{2}+b,\frac{k-\left(rn+p\right)}{2}+l-b\right),\\ \text{if }\frac{k-\left(rl+p-4\right)}{2}\leq q\leq l-\frac{k-\left(rl+p-2\right)}{2}\\ \left(y,y,\frac{m-\left(rl+p+2\right)}{2}+b,k-a\right),\text{if }l-\frac{k-\left(rl+p\right)}{2}\leq b\leq l \end{array} \end{array}$$

These vectors are distinct in at least two coordinates. So *F* is an FTRS for *T*_*k,l*_. Therefore, $\beta^{\prime }\left(T_{k,l}\right)\leq 4$.

**Theorem 2.2**. Let *T*_*k,l*_ denote the graph of *k* × *l*, 2D-lattice of triangular boron nano tubes. Then $\beta^{\prime }\left(T_{k,l}\right)>3$.

*Proof*. For the lower bound on $\beta^{\prime }\left(T_{k,l}\right)$, we discuss the following cases:

**Case I:** *k* ≤ *l*.

We claim that any set of cardinality 3 is not an FTRS for *T*_*k,l*_. Let *F* = {*v*_*i,j*_, *v*_*a,b*_, *v*_*r,s*_} where *i* < *a* < *r* and *j* < *b* < *s* be an FTRS for *T*_*k,l*_. Then *F*_1_ = *F*\{*v*} is a R.S. for each *v* ∈ *F*. We discuss the following possibilities:

**Possibility 1**: When all vertices in *F* lie on the same row.

(i) If all vertices in *F* lie in the first row, then *i* = *a* = *r* = 1. Let *F*_1_ = {*v*_1,*j*_, *v*_1,*b*_} then *r*(*v*_1,*b*+1_|*F*_1_) = *r*(*v*_2,*b*+1_|*F*_1_), a contradiction.

(ii) If all vertices in *F* lie in *p* − *th* row and 1 < *p*. Let *F*_1_ = {*v*_*p,j*_, *v*_*p,b*_} then either *r*(*v*_*k,b*+1_|*F*_1_) = *r*(*v*_*k*−1,*b*+1_|*F*_1_) or *r*(*v*_*p,b*+1_|*F*_1_) = *r*(*v*_*p*+1,*b*+1_|*F*_1_), a contradiction.

**Possibility 2**: When two vertices in *F* lie on the same row.

WLOG we may suppose that *i* = *a* = *l* and *r* ≠ *l*. Let *F*_1_ = {*v*_*l,j*_, *v*_*l,q*_} then either *r*(*v*_*l,q*+1_|*F*_1_) = *r*(*v*_*l*−1,*q*+1_|*F*_1_) or *r*(*v*_*l,q*+1_|*F*_1_) = *r*(*v*_*l*+1,*q*+1_|*F*_1_), a contradiction.

**Possibility 3**: When the vertices in *F* lie on three different rows.

(i) Two vertices in *F* lie on same column, let *j* = *b*. If *j* = *b* = 1 and *F*_1_ = {*v*_1,1_, *v*_*a*,1_} then *r*(*v*_2,1_|*F*_1_) = *r*(*v*_2,2_|*F*_1_), a contradiction.

(ii) If *F*_1_ = {*v*_*i*,1_, *v*_*j*,1_}, 1 ≤ *i* < *j* < *k* then either *r*(*v*_*j*,3_|*F*_1_) = *r*(*v*_*j*+1,3_|*F*_1_) or *r*(*v*_*j*,3_|*F*_1_) = *r*(*v*_*j*+1,2_|*F*_1_), a contradiction.

(iii) If *F*_1_ = {*v*_*i,b*_, *v*_*j,b*_}, 1 ≤ *i* < *j* < *k*, and 1 < *q* < *l* then either *r*(*v*_*j*+1,*b*_|*F*_1_) = *r*(*v*_*j*+1,*b*+1_|*F*_1_) or *r*(*v*_*j*+1,*b*_|*F*_1_) = *r*(*v*_*j*+1,*b*−1_|*F*_1_), a contradiction.

(iv) If all vertices in *S* lie on different columns. Let *S*_1_ = {*V*_*i,j*_, *V*_*a,b*_}, *i* < *a* then

(a) If *j* < *b* then either *r*(*v*_*a,b*+1_|*F*_1_) = *r*(*v*_*a* + 1, *b*+1_|*F*_1_) or *r*(*v*_*a,b*+1_|*F*_1_) = *r*(*v*_*a* + 1, *b*_|*F*_1_), a contradiction.

(b) If *j* > *b* then either *r*(*v*_*a*−1,*b*_|*F*_1_) = *r*(*v*_*a,b*+1_|*F*_1_) or *r*(*v*_*a*−1,*b*+1_|*F*_1_) = *r*(*v*_*a,b*+1_|*F*_1_), a contradiction.

From the above discussion, we conclude that *F* is not an FTRS for *T*_*m,n*_. So $\beta^{\prime }\left(T_{m,n}\right)>3$ in this case. Hence $\beta^{\prime }\left(T_{k,l}\right)=4$.

**Case II:** *k* > *l*

In order to prove that there is no FTRS for *T*_*k,l*_ having cardinality 3, it is enough to prove that any set of vertices having two elements is not an R.S. for *T*_*k,l*_. Let *F*_1_ = {*v*_*i,j*_, *v*_*a,b*_} be an R.S. for *T*_*k,l*_. There are three possibilities

**Possibility 1**: If *v*_*i,j*_, *v*_*a,b*_ are taken from same row, i.e., *i* = *a* then

(i) If *F*_1_ = {*v*_1,1_, *v*_1,*l*_} then $r\left(v_{l+1,\frac{l}{2}}|F_{1}\right)=r\left(v_{l+1,\frac{l+2}{2}}|F_{1}\right)$ if *l* is even and $r\left(v_{l+1,\frac{l+1}{2}}|F_{1}\right)=r\left(v_{l+1,\frac{l+3}{2}}|F_{1}\right)$ if *l* is odd, a contradiction.

(ii) If *F*_1_ = {*v*_*i,j*_, *v*_*i,b*_}, 1 ≤ *i* < *k*, and 1 ≤ *j* < *b* < *l* then *r*(*v*_*i,b*+1_|*F*_1_) = *r*(*v*_*i* + 1, *b*+1_|*F*_1_), a contradiction.

(iii) If *F*_1_ = {*v*_*i,j*_, *v*_*i,b*_}, 1 ≤ *i* < *k*, and 1 < *j* < *b* = *l* then *r*(*v*_*i,j*−1_|*F*_1_) = *r*(*v*_*i* + 1, *j*_|*F*_1_), a contradiction.

(iv) If *F*_1_ = {*v*_*k,j*_, *v*_*k,b*_} and 1 ≤ *j* < *b* < *l* then *r*(*v*_*k,b*+1_|*F*_1_) = *r*(*v*_*k*−1,*b*+1_|*F*_1_) or *r*(*v*_*k,b*+1_|*F*_1_) = *r*(*v*_*k*−1,*b*_|*F*_1_), a contradiction.

(v) If *F*_1_ = {*v*_*k,j*_, *v*_*k,b*_} and 1 < *j* < *b* = *l* then *r*(*v*_*k,j*−1_|*F*_1_) = *r*(*v*_*k*−1,*j*_|*F*_1_), a contradiction.

(vi) If *F*_1_ = {*v*_*i*,1_, *v*_*i,l*_} and 1 < *i* < *k* then *r*(*v*_*i*−1,1_|*F*_1_) = *r*(*v*_*i*+1,1_|*F*_1_), a contradiction.

(vii) If *F*_1_ = {*v*_*k*,1_, *v*_*k,l*_} then *r*(*v*_*k* − *l*, 3_|*F*_1_) = *r*(*v*_*k* − *l*, 4_|*F*_1_), a contradiction.

**Possibility 2**: If *v*_*i,j*_, *v*_*a,b*_ are taken from same column, i.e., *j* = *b* then

(i) If *F*_1_ = {*v*_1,1_, *v*_*k*,1_} then *r*(*v*_2,1_|*F*_1_) = *r*(*v*_2,2_|*F*_1_), a contradiction.

(ii) If *F*_1_ = {*v*_*i*,1_, *v*_*j*,1_} where 1 ≤ *i* < *j* < *m* then *r*(*v*_*j*,3_|*F*_1_) = *r*(*v*_*j*+1,3_|*F*_1_) or *r*(*v*_*j*,3_|*F*_1_) = *r*(*v*_*j*+1,2_|*F*_1_), a contradiction.

(iii) If *F*_1_ = {*v*_*i*,1_, *v*_*j*,1_} and 1 < *i* < *j* = *k* then *r*(*v*_*i*,3_|*F*_1_) = *r*(*v*_*i*−1,2_|*F*_1_) or *r*(*v*_*i*,3_|*F*_1_) = *r*(*v*_*i*−1,3_|*F*_1_), a contradiction.

(iv) If *F*_1_ = {*v*_*i,b*_, *v*_*j,b*_}, 1 ≤ *i* < *j* < *k*, and 1 < *b* < *l* then *r*(*v*_*j*+1,*b*_|*F*_1_) = *r*(*v*_*j*+1,*b*+1_|*F*_1_) or *r*(*V*_*j*+1,*b*_|*S*_1_) = *r*(*V*_*j*+1,*b*−1_|*S*_1_), a contradiction.

(v) If *F*_1_ = {*v*_*i,b*_, *v*_*j,b*_}, 1 < *i* < *j* ≤ *k*, and 1 < *b* < *l* then *r*(*v*_*i*−1,*j*−1_|*F*_1_) = *r*(*v*_*i*−1,*j*_|*F*_1_) or *r*(*v*_*i*−1,*j*_|*F*_1_) = *r*(*v*_*i*−1,*j*+1_|*F*_1_), a contradiction.

(vi) If *F*_1_ = {*v*_*i,b*_, *v*_*j,b*_}, *i* = 1, *j* = *k*, and 1 < *q* < *l* then *r*(*v*_2,*j*_|*F*_1_) = *r*(*v*_2,*j*+1_|*F*_1_), a contradiction.

(vii) If *F*_1_ = {*v*_*i,l*−1_, *v*_*j,l*−1_} and *i* < *j* < *k* then *r*(*v*_*j,l*−2_|*F*_1_) = *r*(*v*_*j*+1,*l* − 2_|*F*_1_) or *r*(*v*_*j,l*−3_|*F*_1_) = *r*(*v*_*j*+1,*l* − 2_|*F*_1_), a contradiction.

(viii) If *F*_1_ = {*v*_1,*l*_, *v*_*k,l*_} then *r*(*v*_*k*−1,*l*−1_|*F*_1_) = *r*(*v*_*k*−1,*l*_|*F*_1_) or *r*(*v*_*k*−2,*l*−1_|*F*_1_) = *r*(*v*_*k*−2,*l*_|*F*_1_), a contradiction.

**Possibility 3**: If *v*_*i,j*_, *v*_*a,b*_ are taken from different rows and different columns, i.e., *i* ≠ *p*,*j* ≠ *q*. Let *F*_1_ = {*v*_*i,j*_, *v*_*a,b*_} and *i* < *a*

(i) If *j* < *b* then *r*(*v*_*a,b*+1_|*F*_1_) = *r*(*v*_*a* + 1, *b*+1_|*F*_1_) or *r*(*v*_*a,b*+1_|*F*_1_) = *r*(*v*_*a* + 1, *b*_|*F*_1_), a contradiction.

(ii) If *j* > *b* then *r*(*v*_*a*−1,*b*_|*F*_1_) = *r*(*v*_*a,b*+1_|*F*_1_) or *r*(*v*_*a*−1,*b*+1_|*F*_1_) = *r*(*v*_*a,b*+1_|*F*_1_), a contradiction.

(iii) If *a* = *k* and *i* = 1 then *r*(*v*_2,*j*_|*F*_1_) = *r*(*v*_2,*j*+1_|*F*_1_) or *r*(*v*_1,*j*+1_|*F*_1_) = *r*(*v*_2,*j*+1_|*F*_1_) or *r*(*v*_*k*−1,*b*+1_|*F*_1_) = *r*(*v*_*k,b*−1_|*F*_1_), a contradiction.

Thus no set having two vertices is an R.S. for *T*_*k,l*_. So $\beta^{\prime }\left(T_{k,l}\right)>3$ in this case. Hence $\beta^{\prime }\left(T_{k,l}\right)=4$.

The next theorem gives $\beta^{\prime }\left(\alpha_{k,l}\right)$.

**Theorem 2.3**. Let α_*k,l*_ denote the graph of *k* × *l* 2D-lattice of α boron nano tubes. Then $\beta^{\prime }\left(\alpha_{k,l}\right)=4$

**Figure 4:**
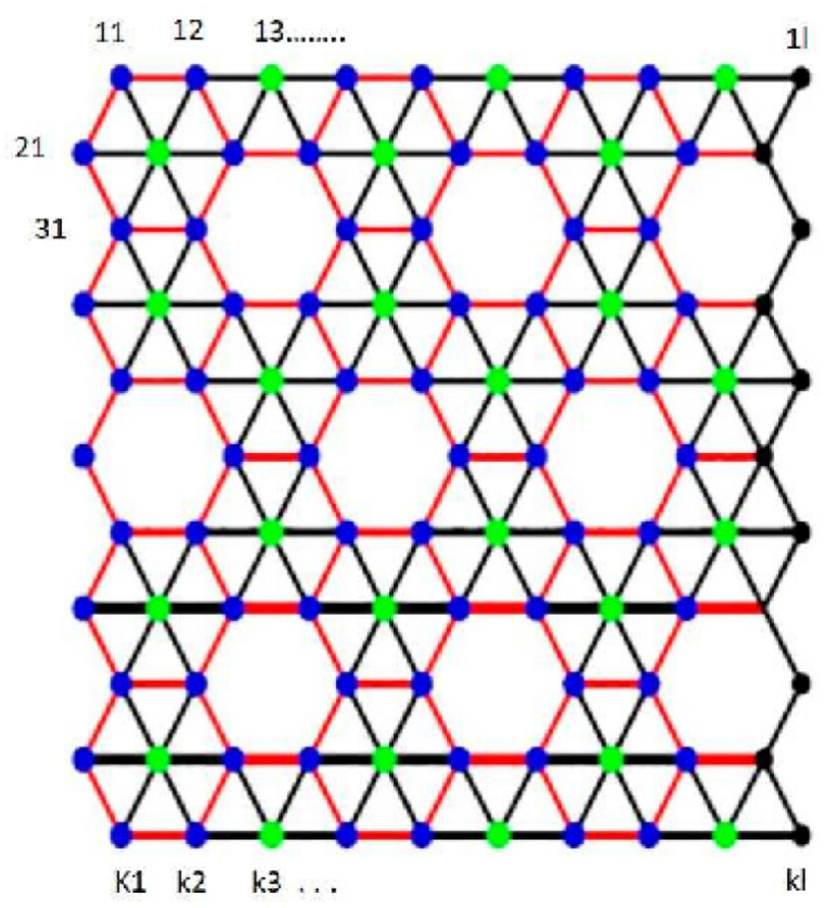
Labeling of vertices of α_*k,l*_.

*Proof*. Consider *k* × *l* 2D-lattice of α-boron Nanotubes. We denote this graph by α_*k,l*_. In general, the tube is “cut” vertically and the upper left triangle is pointing up as shown in Figure 4. This figure presents labeling of vertices that becomes optimal for the basis of fault tolerance. The vertex set of α_*k,l*_ is partitioned as {*v*_1,1_, *v*_1,2_, *v*_1,3_, ...., *v*_1,*l*_, *v*_2,1_, *v*_2,2_, ...., *v*_2,*l*_, *v*_4,1_, *v*_4,2_, ......, *v*_4,*l*_, *v*_5,1_, *v*_5,2_, ......, *v*_5,*l*_, *v*_7,1_, *v*_7,2_, ......, *v*_7,*l*_, ......, *v*_*k*,1_, *v*_*k*,2_, *v*_*k*,3_, ...., *v*_*k,l*_} ∪ {*v*_3,1_, *v*_3,2_, *v*_3,4_, *v*_3,5_, *v*_3,7_, *v*_3,8_, ...., *v*_3,*l*_, *v*_6,1_, *v*_6,3_, *v*_6,4_, *v*_6,6_, *v*_6,7_, *v*_6,9_, ...., *v*_6,*l*_, *v*_9,1_, *v*_9,2_, *v*_94_,, *v*_9,5_, *v*_9,7_, *v*_9,8_, ....,_9,*l*_, ......}.

**Case I: When** *k* < *l*.

Let *F* = {*v*_1,1_, *v*_1,*l*_, *v*_*k*,1_, *v*_*k,l*_}. We show that *F* is an FTRS for α_*k,l*_. We give the distance vectors of all vertices *v*_*a,b*_ of α_*k,l*_ relative to *F*.

Let *k* be odd and *k* ≠ 6*q* + 1, *q* ∈ *Z*^+^.

For *a* = 1


$$\begin{array}{l} \;r\left(v_{a,b}|S\right)\\ =\{\begin{array}{c} \left(b-1,l-b,m-1,l+\frac{k-a}{2}-a\right),\text{if }1\leq b\leq \frac{k+1}{2}\\ \left(b-1,l-b,b+\frac{k-3}{2},l+\frac{k-a}{2}-b\right),\text{if }\frac{k+3}{2}\leq b\leq l-\frac{k+1}{2}\\ \left(b-1,l-b,b+\frac{k-3}{2},k-1\right),\text{if }l-\frac{k-1}{2}\leq b\leq l \end{array} \end{array}$$

For *a* = 2


$$\begin{array}{l} \;r\left(v_{a,b}|S\right)\\ =\{\begin{array}{c} \left(1,l,k-2,l+\frac{x+1}{2}-b\right),\text{if }b=1\\ \left(b-1,1+l-b,k-2,l+\frac{x+1}{2}-b\right),\text{if }2\leq b\leq \frac{k+1}{2}\\ \left(b-1,1+l-b,b+\frac{k-5}{2},l+\frac{x+1}{2}-b\right),\text{if }\frac{k+3}{2}\\ \leq b\leq l-\frac{k-1}{2}\\ \left(b-1,1+l-b,b+\frac{k-5}{2},k-2\right),\text{if }l-\frac{k-3}{2}\leq b\leq l \end{array} \end{array}$$

In general, if *a* is odd, *a* ≠ 3*p*, *p* ∈ *Z*^+^, and $3\leq a\leq \left\lceil \frac{k}{2}\right\rceil$ then


$$\begin{array}{l} \;r\left(v_{a,b}|S\right)\\ =\{\begin{array}{c} \left(a-1,l+\left\lfloor \frac{a}{2}\right\rfloor -b,x,l+\frac{x}{2}-b\right),\text{if }1\leq b\leq \frac{a-1}{2}\\ \left(\left\lfloor \frac{a-3}{2}\right\rfloor +b,l+\left\lfloor \frac{a}{2}\right\rfloor -b,x,l+\frac{x}{2}-b\right),\text{if }\frac{a+1}{2}\\ \leq b\leq l-\frac{x+2}{2}\\ \left(\left\lfloor \frac{a-3}{2}\right\rfloor +b,l+\left\lfloor \frac{a}{2}\right\rfloor -b,x,x\right),\text{if }l-\frac{x}{2}\\ \leq b\leq \frac{m-\left(a-2\right)}{2}\\ \left(\left\lfloor \frac{a-3}{2}\right\rfloor +b,l+\left\lfloor \frac{a}{2}\right\rfloor -b,b+\frac{x-2}{2},k-a\right),\\ \text{if }\frac{x+4}{2}\leq b\leq l-\frac{a-1}{2}\\ \left(\left\lfloor \frac{a-3}{2}\right\rfloor +b,a-1,b+\frac{x-2}{2},x\right),\text{if }l-\frac{a-3}{2}\leq b\leq l \end{array} \end{array}$$

If *a* is odd, *a* ≠ 3*p*, *p* ∈ *Z*^+^, and $\left\lceil \frac{k}{2}\right\rceil +1\leq a\leq k$ then


$$\begin{array}{l} \;r\left(v_{a,b}|S\right)\\ =\{\begin{array}{c} \left(a-1,l+\left\lfloor \frac{a}{2}\right\rfloor -b,x,\frac{x}{2}+l-b\right),\text{if }1\leq b\leq \frac{x+2}{2}\\ \left(a-1,l+\left\lfloor \frac{a}{2}\right\rfloor -b,\frac{x-2}{2}+b,\frac{x}{2}+l-b\right),\text{if }\frac{x+4}{2}\\ \leq b\leq l-\frac{a-1}{2}\\ \left(a-1,a-1,b+\frac{x-2}{2},\frac{x}{2}+l-b\right),\text{if }l-\frac{a-3}{2}\leq b\leq \frac{a-1}{2}\\ \left(\left\lfloor \frac{a-3}{2}\right\rfloor +b,a-1,b+\frac{x-2}{2},\frac{x}{2}-b+l\right),\text{if }\frac{a+1}{2}\\ \leq b\leq l-\frac{x+2}{2}\\ \left(\left\lfloor \frac{a-3}{2}\right\rfloor +b,a-1,b+\frac{x-2}{2},x\right),\text{if }l-\frac{x}{2}\leq b\leq l \end{array} \end{array}$$

If *a* is even, *a* ≠ 3*p*, *p* ∈ *Z*^+^, and $3<a\leq \left\lceil \frac{k}{2}\right\rceil$ then


$$\begin{array}{l} \;r\left(v_{a,b}|S\right)\\ =\{\begin{array}{c} \left(a-1,l+\left\lfloor \frac{a}{2}\right\rfloor -b,x,\frac{x+1}{2}+l-b\right),\text{if }1\leq b\leq \frac{a}{2}\\ \left(b+\left\lfloor \frac{a-3}{2}\right\rfloor ,l+\left\lfloor \frac{a}{2}\right\rfloor -b,x,\frac{x+1}{2}+l-b\right),\text{if }\frac{a+2}{2}\\ \leq b\leq \frac{x+3}{2}\\ \left(b+\left\lfloor \frac{a-3}{2}\right\rfloor ,l+\left\lfloor \frac{a}{2}\right\rfloor -b,x,x\right),\text{if }\frac{x+5}{2}\leq b\leq l-\frac{x+1}{2}\\ \left(\left\lfloor \frac{a-3}{2}\right\rfloor +b,l+\left\lfloor \frac{a}{2}\right\rfloor -b,b+\frac{x-3}{2},k-a\right),\text{if }l-\frac{x-1}{2}\\ \leq b\leq l-\frac{a-2}{2}\\ \left(\left\lfloor \frac{a-3}{2}\right\rfloor +b,a-1,\frac{x-3}{2}+b,x\right),\text{if }l-\frac{a-4}{2}\leq b\leq l \end{array} \end{array}$$

If *a* is even, *a* ≠ 3*p*, *p* ∈ *Z*^+^, and $\left\lceil \frac{k}{2}\right\rceil +1\leq a\leq k$ then


$$\begin{array}{l} \;r\left(v_{a,b}|S\right)\\ =\{\begin{array}{c} \left(a-1,l+\left\lfloor \frac{a}{2}\right\rfloor -b,k-a,\frac{x+1}{2}+l-b\right),\text{if }1\leq b\leq \frac{x+1}{2}\\ \left(a-1,l+\left\lfloor \frac{a}{2}\right\rfloor -b,q+\frac{x-3}{2},\frac{x+1}{2}+l-b\right),\text{if }\frac{x+3}{2}\\ \leq b\leq \frac{a}{2}\\ \left(\left\lfloor \frac{a-3}{2}\right\rfloor +b,l+\left\lfloor \frac{a}{2}\right\rfloor -b,b+\frac{x-3}{2},\frac{x+1}{2}+l-b\right),\\ \text{if }\frac{a+2}{2}\leq b\leq l-\frac{a}{2}\\ \left(\left\lfloor \frac{a-3}{2}\right\rfloor +b,a-1,\frac{x-3}{2}+b,\frac{x+1}{2}+l-b\right),\text{if }l-\frac{a-2}{2}\\ \leq b\leq l-\frac{x+1}{2}\\ \left(\left\lfloor \frac{a-3}{2}\right\rfloor +b,a-1,\frac{x-3}{2}+b,x\right),\text{if }l-\frac{x-1}{2}\leq b\leq l \end{array} \end{array}$$

Let *k* = 6*p* + 1.

If *a* is odd, *a* ≠ 3*p*, *k* ∈ *Z*^+^, and $3<a\leq \left\lceil \frac{k}{2}\right\rceil$ then


$$\begin{array}{l} \;r\left(v_{a,b}|S\right)\\ =\{\begin{array}{c} \left(a-1,l+\left\lfloor \frac{a}{2}\right\rfloor -b,x,\frac{x}{2}-b+l\right),\text{if }1\leq b\leq \frac{a-1}{2}\\ \left(\left\lfloor \frac{a-3}{2}\right\rfloor +b,l+\left\lfloor \frac{a}{2}\right\rfloor -b,x,\frac{x}{2}-b+l\right),\text{if }\frac{a+1)}{2}\\ \leq b\leq l-\frac{x+2}{2}\\ \left(\left\lfloor \frac{a-3}{2}\right\rfloor +b,l+\left\lfloor \frac{a}{2}\right\rfloor -b,x,x\right),\text{if }l-\frac{x}{2}\leq b\leq \frac{x}{2}\\ \left(\left\lfloor \frac{a-3}{2}\right\rfloor +b,l+\left\lfloor \frac{a}{2}\right\rfloor -b,x+1,x\right),\text{if }b=\frac{x+2}{2}\\ \left(\left\lfloor \frac{a-3}{2}\right\rfloor +b,\left\lfloor \frac{a}{2}\right\rfloor -b+l,b+\frac{x-2}{2},x\right),\\ \text{if }\frac{x+4}{2}\leq b\leq l-\frac{a-1}{2}\\ \left(\left\lfloor \frac{a-3}{2}\right\rfloor +b,a-1,\frac{x-2}{2}+b,x\right),\text{if }l-\frac{a-3}{2}\leq b\leq l \end{array} \end{array}$$

If *a* is odd, *a* ≠ 3*p*, *p* ∈ *Z*^+^, and $\left\lceil \frac{k}{2}\right\rceil +1\leq a\leq k$ then


$$\begin{array}{l} \;r\left(v_{a,b}|S\right)\\ =\{\begin{array}{c} \left(a-1,l+\left\lfloor \frac{a}{2}\right\rfloor -b,x,\frac{x}{2}-b+l\right),\text{if }1\leq b\leq \frac{x}{2}\\ \left(a-1,l+\left\lfloor \frac{a}{2}\right\rfloor -b,x+1,\frac{x}{2}-b+l\right),\text{if }b=\frac{x+2}{2}\\ \left(a-1,l+\left\lfloor \frac{a}{2}\right\rfloor -b,b+\frac{x-2}{2},\frac{x}{2}-b+l\right),\text{if }\frac{x+4}{2}\\ \leq b\leq l-\frac{a-1}{2}\\ \left(a-1,a-1,b+\frac{x-2}{2},\frac{x}{2}-b+l\right),\text{if }l-\frac{a-3}{2}\leq b\leq \frac{a-1}{2}\\ \left(\left\lfloor \frac{a-3}{2}\right\rfloor +b,a-1,\frac{x-2}{2}+b,\frac{x}{2}-b+l\right),\\ \text{if }\frac{a+1}{2}\leq b\leq l-\frac{x+2}{2}\\ \left(\left\lfloor \frac{a-3}{2}\right\rfloor +b,a-1,\frac{x-2}{2}+b,x\right),\text{if }l-\frac{x}{2}\leq b\leq k \end{array} \end{array}$$

If *a* is even, *a* ≠ 3*p*, *p* ∈ *Z*^+^, and $3<a\leq \left\lceil \frac{k}{2}\right\rceil$ then


$$\begin{array}{l} \;r\left(v_{a,b}|S\right)\\ =\{\begin{array}{c} \left(a-1,\left\lfloor \frac{a}{2}\right\rfloor -b+l,x,\frac{x+1}{2}-b+l\right),\text{if }1\leq b\leq \frac{a}{2}\\ \left(\left\lfloor \frac{a-3}{2}\right\rfloor +b,\left\lfloor \frac{a}{2}\right\rfloor -b+l,x,\frac{x+1}{2}-b+l\right),\text{if }\frac{a+2}{2}\\ \leq b\leq \frac{x+1}{2}\\ \left(\left\lfloor \frac{a-3}{2}\right\rfloor +b,\left\lfloor \frac{a}{2}\right\rfloor -b+l,x+1,\frac{x+1}{2}-b+l\right),\text{if }b=\frac{x+3}{2}\\ \left(\left\lfloor \frac{a-3}{2}\right\rfloor +b,\left\lfloor \frac{a}{2}\right\rfloor -b+l,x,x\right),\text{if }\frac{x+5}{2}\\ \leq b\leq l-\frac{x+1}{2}\\ \left(\left\lfloor \frac{a-3}{2}\right\rfloor +b,\left\lfloor \frac{a}{2}\right\rfloor -b+l,b+\frac{x-3}{2},k-a\right),\text{if }l-\frac{x-1}{2}\\ \leq b\leq l-\frac{a-2}{2}\\ \left(\left\lfloor \frac{a-3}{2}\right\rfloor +b,a-1,b+\frac{x-3}{2},x\right),\text{if }l-\frac{a-4}{2}\leq b\leq l \end{array} \end{array}$$

If *a* is even, *a* ≠ 3*p*, *p* ∈ *Z*^+^, and $\left\lceil \frac{k}{2}\right\rceil +1\leq a\leq k$ then


$$\begin{array}{l} \;r\left(v_{a,b}|S\right)\\ =\{\begin{array}{c} \left(a-1,l+\left\lfloor \frac{a}{2}\right\rfloor -b,x,\frac{x+1}{2}-b+l\right),\text{if }1\leq b\leq \frac{x+1)}{2}\\ \left(a-1,l+\left\lfloor \frac{a}{2}\right\rfloor -b,x+1,\frac{x+1}{2}-b+l\right),\text{if }b=\frac{x+3}{2}\\ \left(a-1,l+\left\lfloor \frac{a}{2}\right\rfloor -b,b+\frac{x-3}{2},\frac{x+1}{2}-b+l\right),\text{if }\frac{x+5)}{2}\\ \leq b\leq \frac{a}{2}\\ \left(b+\left\lfloor \frac{a-3}{2}\right\rfloor ,l+\left\lfloor \frac{a}{2}\right\rfloor -b,b+\frac{x-3}{2},\frac{x+1}{2}-b+l\right),\text{if }\frac{a+2}{2}\\ \leq b\leq l-\frac{a}{2}\\ \left(\left\lfloor \frac{a-3}{2}\right\rfloor +b,a-1,\frac{x-3}{2}+b,\frac{x+1}{2}-b+l\right),\text{if }l-\frac{a-2}{2}\\ \leq b\leq l-\frac{x+1}{2}\\ \left(\left\lfloor \frac{a-3}{2}\right\rfloor +b,a-1,\frac{x-3}{2}+b,x\right),\text{if }l-\frac{x-1}{2}\leq b\leq l \end{array} \end{array}$$

If *a* is odd, *a* = 3*p*, *p* ∈ *Z*^+^, $3\leq a\leq \left\lceil \frac{k}{2}\right\rceil$, and *b* ≠ 3*q* then


$$\begin{array}{l} \;r\left(va,b|S\right)\\ =\{\begin{array}{c} \left(a-1,\left\lfloor \frac{a}{2}\right\rfloor -b+l,x,\frac{x}{2}-b+l\right),\text{if }1\leq b\leq \frac{a-1}{2}\\ \left(b+\left\lfloor \frac{a-3}{2}\right\rfloor ,\left\lfloor \frac{a}{2}\right\rfloor -b+l,x,\frac{x}{2}-b+l\right),\text{if }\frac{a+1}{2}\\ \leq b\leq l-\frac{x+2}{2}\\ \left(b+\left\lfloor \frac{a-3}{2}\right\rfloor ,l+\left\lfloor \frac{a}{2}\right\rfloor -b,x,x\right),\text{if }l-\frac{x}{2}\\ \leq b\leq \frac{x+2}{2}\\ \left(b+\left\lfloor \frac{a-3}{2}\right\rfloor ,l+\left\lfloor \frac{a}{2}\right\rfloor -b,\frac{x-2}{2}+b,x\right),\text{if }\frac{x+4}{2}\\ \leq b\leq l-\frac{a-1}{2}\\ \left(\left\lfloor \frac{a-3}{2}\right\rfloor +b,a-1,b+\frac{x-2}{2},x\right),\text{if }l-\frac{a-3}{2}\leq b\leq l \end{array} \end{array}$$

If *a* is odd, *a* = 3*p*, *p* ∈ *Z*^+^, $\left\lceil \frac{k}{2}\right\rceil +1\leq a\leq k$, and *b* ≠ 3*q* then


$$\begin{array}{l} \;r\left(v_{a,b}|S\right)\\ =\{\begin{array}{c} \left(a-1,l+\left\lfloor \frac{a}{2}\right\rfloor -b,x,l+\frac{x}{2}-b\right),\text{if }1\leq b\leq \frac{x+2}{2}\\ \left(a-1,\left\lfloor \frac{a}{2}\right\rfloor -b+l,\frac{x-2}{2}+b,\frac{x}{2}-b+l\right),\text{if }\frac{x+4}{2}\\ \leq b\leq l-\frac{a-1}{2}\\ \left(a-1,a-1,b+\frac{x-2}{2},l+\frac{k-a}{2}-b\right),\text{if }l-\frac{a-3}{2}\\ \leq b\leq \frac{a-1}{2}\\ \left(\left\lfloor \frac{a-3}{2}\right\rfloor +b,a-1,\frac{x-2}{2}+b,\frac{x}{2}-b+l\right),\text{if }\frac{a+1}{2}\\ \leq b\leq l-\frac{x+2}{2}\\ \left(\left\lfloor \frac{a-3}{2}\right\rfloor +b,a-1,\frac{x-2}{2}+b,x\right),\text{if }l-\frac{x}{2}\leq b\leq l \end{array} \end{array}$$

If *a* is even, *a* = 3*p*, *p* ∈ *Z*^+^, $3<a\leq \left\lceil \frac{k}{2}\right\rceil$, and *b* ≠ 3*q* − 1 then


$$\begin{array}{l} \;r\left(v_{a,b}|S\right)\\ =\{\begin{array}{c} \left(a-1,l+\left\lfloor \frac{a}{2}\right\rfloor -b,x,\frac{x+1}{2}-b+l\right),\text{if }1\leq b\leq \frac{a}{2},\\ b\neq 3l-1\\ \left(b+\left\lfloor \frac{a-3}{2}\right\rfloor ,l+\left\lfloor \frac{a}{2}\right\rfloor -b,x,l+\frac{x+1}{2}-b\right),\text{if }\frac{a+2}{2}\\ \leq b\leq \frac{x+3}{2}\\ \left(b+\left\lfloor \frac{a-3}{2}\right\rfloor ,\left\lfloor \frac{a}{2}\right\rfloor -b+l,x,x\right),\text{if }\frac{x+5}{2}\leq b\leq l-\frac{x+1}{2}\\ \left(\left\lfloor \frac{a-3}{2}\right\rfloor +b,l+\left\lfloor \frac{a}{2}\right\rfloor -b,b+\frac{x-3}{2},x\right),\text{if }l-\frac{x-1}{2}\\ \leq b\leq l-\frac{a-2}{2}\\ \left(\left\lfloor \frac{a-3}{2}\right\rfloor +b,a-1,\frac{x-3}{2}+b,x\right),\text{if }l-\frac{a-4}{2}\leq b\leq l \end{array} \end{array}$$

If *a* is even, *a* = 3*p*, *p* ∈ *Z*^+^, $\left\lceil \frac{k}{2}\right\rceil +1\leq a\leq k$, and *b* ≠ 3*q* − 1 then


$$\begin{array}{l} \;r\left(v_{a,b}|S\right)\\ =\{\begin{array}{c} \left(a-1,\left\lfloor \frac{a}{2}\right\rfloor -b+l,x,\frac{x+1}{2}-b+l\right),\text{if }1\leq b\leq \frac{x+1)}{2}\\ \left(a-1,\left\lfloor \frac{a}{2}\right\rfloor -b+l,\frac{x-3}{2}+b,\frac{x+1}{2}-b+l\right),\text{if }\frac{x+3}{2}\\ \leq b\leq \frac{a}{2}\\ \left(b+\left\lfloor \frac{a-3}{2}\right\rfloor ,l+\left\lfloor \frac{a}{2}\right\rfloor -b,b+\frac{x-3)}{2},\frac{x+1}{2}-b+l\right),\text{if }\frac{a+2}{2}\\ \leq b\leq l-\frac{a}{2}\\ \left(\left\lfloor \frac{a-3}{2}\right\rfloor +b,a-1,\frac{x-3}{2}+b,\frac{x+1}{2}-b+l\right),\text{if }l-\frac{a-2}{2}\\ \leq b\leq l-\frac{x+1}{2}\\ \left(\left\lfloor \frac{a-3}{2}\right\rfloor +b,a-1,\frac{x-3}{2}+b,x\right),\text{if }l-\frac{x-1}{2}\leq b\leq l \end{array} \end{array}$$

Let *k* be even and *k* ≠ 6*p*+2, *p* ∈ *Z*^+^.

If *a* is odd, *a* ≠ 3*p*, *p* ∈ *Z*^+^, and $3<a\leq \left\lceil \frac{k}{2}\right\rceil$ then


$$\begin{array}{l} \;r\left(v_{a,b}|S\right)\\ =\{\begin{array}{c} \left(a-1,\left\lfloor \frac{a}{2}\right\rfloor -b+l,x,\frac{x}{2}-b+l\right),\text{if }1\leq b\leq \frac{a-1}{2}\\ \left(b+\left\lfloor \frac{a-3}{2}\right\rfloor ,l+\left\lfloor \frac{a}{2}\right\rfloor -b,x,\frac{x-1}{2}-b+l\right),\text{if }\frac{a+1}{2}\\ \leq b\leq l-\frac{x-3}{2}\\ \left(b+\left\lfloor \frac{a-3}{2}\right\rfloor ,\left\lfloor \frac{a}{2}\right\rfloor -b+l,x,x\right),\text{if }n-\frac{x+1}{2}\leq b\leq \frac{x+1}{2}\\ \left(\left\lfloor \frac{a-3}{2}\right\rfloor +b,\left\lfloor \frac{a}{2}\right\rfloor -b+l,b+\frac{x-1)}{2},x\right),\text{if }\frac{x+3)}{2}\\ \leq b\leq l-\frac{a+1}{2}\\ \left(b+\left\lfloor \frac{a-3}{2}\right\rfloor ,a-1,b+\frac{x-1)}{2},x\right),\text{if }l-\frac{a-1}{2}\leq b\leq l \end{array} \end{array}$$

If *a* is odd, *a* ≠ 3*p*, *p* ∈ *Z*^+^, and $\left\lceil \frac{k}{2}\right\rceil +1\leq a\leq k$ then


$$\begin{array}{l} \;r\left(v_{a,b}|S\right)\\ =\{\begin{array}{c} \left(a-1,\left\lfloor \frac{a}{2}\right\rfloor -b+l,x,\frac{x-1}{2}-b+l\right),\text{if }1\leq b\leq \frac{x+1}{2}\\ \left(a-1,\left\lfloor \frac{a}{2}\right\rfloor -b+l,\frac{x-1}{2}+b,\frac{x-1}{2}-b+l\right),\text{if }\frac{x-3}{2}\\ \leq b\leq l-\frac{a+1}{2}\\ \left(a-1,a-1,b+\frac{k-\left(a+1\right)}{2},l+\frac{x-1}{2}-b\right),\text{if }l-\frac{b-1}{2}\\ \leq b\leq \frac{a-1}{2}\\ \left(b+\left\lfloor \frac{a-3}{2}\right\rfloor ,a-1,\frac{x-1)}{2}+b,\frac{x-1}{2}-b+l\right),\text{if }\frac{a+1}{2}\\ \leq b\leq l-\frac{x+3)}{2}\\ \left(b+\left\lfloor \frac{a-3}{2}\right\rfloor ,a-1,b+\frac{x-1)}{2},x\right),\text{if }l-\frac{x+1}{2}\leq b\leq l \end{array} \end{array}$$

If *a* is even, *a* ≠ 3*p*, *p* ∈ *Z*^+^, and $3<a\leq \left\lceil \frac{k}{2}\right\rceil$ then


$$\begin{array}{l} \;r\left(v_{a,b}|S\right)\\ =\{\begin{array}{c} \left(a-1,\left\lfloor \frac{a}{2}\right\rfloor -b+l,x,\frac{x}{2}-b+l\right),\text{if }1\leq b\leq \frac{a}{2}\\ \left(b+\left\lfloor \frac{a-3}{2}\right\rfloor ,\left\lfloor \frac{a}{2}\right\rfloor -b+l,x,\frac{x}{2}-b+l\right),\text{if }\frac{a+2}{2}\\ \leq b\leq l-\frac{x+2}{2}\\ \left(b+\left\lfloor \frac{a-3}{2}\right\rfloor ,l+\left\lfloor \frac{a}{2}\right\rfloor -b,\frac{x-2)}{2}+b,\frac{x}{2}-b+l\right),\text{if }l-\frac{x}{2}\\ \leq b\leq \frac{x+2}{2}\\ \left(b+\left\lfloor \frac{a-3}{2}\right\rfloor ,l+\left\lfloor \frac{a}{2}\right\rfloor -b,\frac{x-2)}{2}+b,x\right),\text{if }\frac{x+4}{2}\\ \leq b\leq l-\frac{a-2}{2}\\ \left(\left\lfloor \frac{a-3}{2}\right\rfloor +b,a-1,b+\frac{x-2}{2},x\right),\text{if }l-\frac{a-4}{2}\leq b\leq l \end{array} \end{array}$$

If *a* is even, *a* ≠ 3*p*, *p* ∈ *Z*^+^, and $\left\lceil \frac{k}{2}\right\rceil +1\leq a\leq k$ then


$$\begin{array}{l} \;r\left(v_{a,b}|S\right)\\ =\{\begin{array}{c} \left(a-1,l+\left\lfloor \frac{a}{2}\right\rfloor -b,x,l+\frac{x}{2}-b\right),\text{if }1\leq b\leq \frac{x+2}{2}\\ \left(a-1,\left\lfloor \frac{a}{2}\right\rfloor -b+l,\frac{x-2}{2}+b,\frac{x}{2}-b+l\right),\text{if }\frac{x+4}{2}\\ \leq b\leq l-\frac{a-2}{2}\\ \left(b+\left\lfloor \frac{a-3}{2}\right\rfloor ,\left\lfloor \frac{a}{2}\right\rfloor -b+l,\frac{x-3}{2}+b,l+\frac{x}{2}-b\right),\text{if }l-\frac{a-4}{2}\\ \leq b\leq \frac{a}{2}\\ \left(\left\lfloor \frac{a-3}{2}\right\rfloor +b,a-1,\frac{x-2}{2}+b,\frac{x}{2}-b+l\right),\text{if }\frac{a+2}{2}\\ \leq b\leq l-\frac{x+2}{2}\\ \left(b+\left\lfloor \frac{a-3}{2}\right\rfloor ,a-1,b+\frac{x-2}{2},x\right),\text{if }l-\frac{x}{2}\leq b\leq l \end{array} \end{array}$$

Let *m* = 6*k*+2.

If *a* is odd, *a* ≠ 3*p*, *p* ∈ *Z*^+^, and $3\leq a\leq \left\lceil \frac{k}{2}\right\rceil$ then


$$\begin{array}{l} \;r\left(v_{a,b}|S\right)\\ =\{\begin{array}{c} \left(a-1,\left\lfloor \frac{a}{2}\right\rfloor -b+l,x,\frac{x-1}{2}-b+l\right),\text{if }1\leq b\leq \frac{a-1}{2}\\ \left(\left\lfloor \frac{a-3}{2}\right\rfloor +b,\left\lfloor \frac{a}{2}\right\rfloor -b+l,x,\frac{x-1)}{2}-b+l\right),\text{if }\frac{a+1)}{2}\\ \leq b\leq l-\frac{x+3}{2}\\ \left(\left\lfloor \frac{a-3}{2}\right\rfloor +b,\left\lfloor \frac{a}{2}\right\rfloor -b+l,x,x\right),\text{if }l-\frac{x+1)}{2}\\ \leq b\leq \frac{x-1}{2}\\ \left(\left\lfloor \frac{a-3}{2}\right\rfloor +b,l+\left\lfloor \frac{a}{2}\right\rfloor -b,x+1,x\right),\text{if }b=\frac{x+1}{2}\\ \left(\left\lfloor \frac{a-3}{2}\right\rfloor +b,\left\lfloor \frac{a}{2}\right\rfloor -b+l,\frac{x-1}{2}+b,x\right),\text{if }\frac{x+3}{2}\\ \leq b\leq l-\frac{a+1}{2}\\ \left(\left\lfloor \frac{a-3}{2}\right\rfloor +b,a-1,\frac{x-1)}{2}+b,x\right),\text{if }l-\frac{a-1}{2}\leq b\leq l \end{array} \end{array}$$

If *a* is odd, *a* ≠ 3*p*, *p* ∈ *Z*^+^, and $\left\lceil \frac{p}{2}\right\rceil +1\leq a\leq p$ then


$$\begin{array}{l} \;r\left(v_{a,b}|S\right)\\ =\{\begin{array}{c} \left(a-1,l+\left\lfloor \frac{a}{2}\right\rfloor -b,x,\frac{x-1}{2}-b+l\right),\text{if }1\leq b\leq \frac{a-1}{2}\\ \left(\left\lfloor \frac{a-3}{2}\right\rfloor +b,\left\lfloor \frac{a}{2}\right\rfloor -b+l,x,\frac{x-1}{2}-b+l\right),\text{if }\frac{a+1}{2}\\ \leq b\leq l-\frac{x+3}{2}\\ \left(\left\lfloor \frac{a-3}{2}\right\rfloor +b,l+\left\lfloor \frac{a}{2}\right\rfloor -b,x,x\right),\text{if }l-\frac{1+x}{2}\leq b\leq \frac{x-1}{2}\\ \left(b+\left\lfloor \frac{a-3}{2}\right\rfloor ,l+\left\lfloor \frac{a}{2}\right\rfloor -b,1+x,x\right),\text{if }b=\frac{1+x)}{2}\\ \left(\left\lfloor \frac{a-3}{2}\right\rfloor +b,\left\lfloor \frac{a}{2}\right\rfloor -b+l,\frac{x-1}{2}+b,x\right),\text{if }\frac{3+x}{2}\\ \leq b\leq l-\frac{a+1}{2}\\ \left(\left\lfloor \frac{a-3}{2}\right\rfloor +b,a-1,\frac{x-1}{2}+b,x\right),\text{if }l-\frac{a-1}{2}\leq b\leq l \end{array} \end{array}$$

If *a* is even, *a* ≠ 3*p*, *p* ∈ *Z*^+^, and $3<a\leq \left\lceil \frac{k}{2}\right\rceil$ then


$$\begin{array}{l} \;r\left(v_{a,b}|S\right)\\ =\{\begin{array}{c} \left(a-1,l+\left\lfloor \frac{a}{2}\right\rfloor -b,x,\frac{1+x}{2}-b+l\right),\text{if }1\leq b\leq \frac{a}{2}\\ \left(\left\lfloor \frac{a-3}{2}\right\rfloor +b,l+\left\lfloor \frac{a}{2}\right\rfloor -b,x,l+\frac{x+1}{2}-b\right),\text{if }\frac{2+a}{2}\\ \leq b\leq \frac{1+x}{2}\\ \left(\left\lfloor \frac{a-3}{2}\right\rfloor +b,\left\lfloor \frac{a}{2}\right\rfloor -b+l,1+x,\frac{x+1)}{2}-b+l\right),\text{if }b=\frac{x+3)}{2}\\ \left(b+\left\lfloor \frac{a-3}{2}\right\rfloor ,\left\lfloor \frac{a}{2}\right\rfloor -b+l,x,x\right),\text{if }\frac{5+x}{2}\\ \leq b\leq l-\frac{1+x)}{2}\\ \left(b+\left\lfloor \frac{a-3}{2}\right\rfloor ,l+\left\lfloor \frac{a}{2}\right\rfloor -b,b+\frac{x-3}{2},x\right),\text{if }l-\frac{x-1}{2}\\ \leq b\leq l-\frac{a-2}{2}\\ \left(\left\lfloor \frac{a-3}{2}\right\rfloor +b,a-1,b+\frac{x-3}{2},x\right),\text{if }l-\frac{a-4}{2}\leq b\leq l \end{array} \end{array}$$

If *a* is even, *a* ≠ 3*p*, *p* ∈ *Z*^+^, and $\left\lceil \frac{k}{2}\right\rceil +1\leq p\leq k$ then


$$\begin{array}{l} \;r\left(v_{a,b}|S\right)\\ =\{\begin{array}{c} \left(a-1,l+\left\lfloor \frac{a}{2}\right\rfloor -b,x,l+\frac{x}{2}-b\right),\text{if }1\leq b\leq \frac{x}{2}\\ \left(a-1,l+\left\lfloor \frac{a}{2}\right\rfloor -b,x+1,l+\frac{x}{2}-b\right),\text{if }b=\frac{x+2}{2}\\ \left(a-1,\left\lfloor \frac{a}{2}\right\rfloor -b+l,b+\frac{x-2}{2},\frac{x}{2}-b+l\right),\text{if }\frac{x+4}{2}\\ \leq b\leq l-\frac{a-2}{2}\\ \left(a-1,a-1,b+\frac{x-2}{2},l+\frac{x}{2}-b\right),\text{if }l-\frac{a-4}{2}\leq b\leq \frac{a}{2}\\ \left(\left\lfloor \frac{a-3}{2}\right\rfloor +b,a-1,\frac{x-2}{2}+b,l+\frac{x}{2}-b\right),\text{if }l-\frac{a-2}{2}\\ \leq b\leq l-\frac{x+1}{2}\\ \left(b+\left\lfloor \frac{a-3}{2}\right\rfloor ,a-1,b+\frac{x-2)}{2},x\right),\text{if }l-\frac{x}{2}\leq b\leq l \end{array} \end{array}$$

If *a* is odd, *a* = 3*p*, *p* ∈ *Z*^+^, $3\leq a\leq \left\lceil \frac{k}{2}\right\rceil$, *b* ≠ 3*q*, and *q* ∈ *Z*^+^ then


$$\begin{array}{l} \;r\left(v_{a,b}|S\right)\\ =\{\begin{array}{c} \left(a-1,\left\lfloor \frac{a}{2}\right\rfloor -b+l,x,\frac{x}{2}-b+l\right),\text{if }1\leq b\leq \frac{x}{2}\\ \left(a-1,\left\lfloor \frac{a}{2}\right\rfloor -b+l,1+x,\frac{x}{2}-b+l\right),\text{if }b=\frac{x+2}{2}\\ \left(a-1,\left\lfloor \frac{a}{2}\right\rfloor -b+l,\frac{x-2}{2}+b,\frac{x}{2}-b+l\right),\text{if }\frac{x+4}{2}\\ \leq b\leq l-\frac{a-2}{2}\\ \left(a-1,a-1,\frac{x-2)}{2}+b,\frac{x}{2}-b+l\right),\text{if }l-\frac{a-4}{2}\leq b\leq \frac{a}{2}\\ \left(b+\left\lfloor \frac{a-3}{2}\right\rfloor ,a-1,\frac{x-2)}{2}+b,\frac{x}{2}-b+l\right),\text{if }\frac{2+a}{2}\\ \leq b\leq l-\frac{m-\left(a-2\right)}{2}\\ \left(b+\left\lfloor \frac{a-3}{2}\right\rfloor ,a-1,\frac{x-2)}{2}+b,x\right),\text{if }l-\frac{x}{2}\leq b\leq l \end{array} \end{array}$$

If *a* is odd, *a* = 3*p*, *p* ∈ *Z*^+^, $\left\lceil \frac{k}{2}\right\rceil +1\leq a\leq k$, *b* ≠ 3*q*, and *q* ∈ *Z*^+^ then


$$\begin{array}{l} \;r\left(v_{a,b}|S\right)\\ =\{\begin{array}{c} \left(a-1,l+\left\lfloor \frac{a}{2}\right\rfloor -b,x,\frac{x-1}{2}-b+l\right),\text{if }1\leq b\leq \frac{x-1}{2}\\ \left(a-1,l+\left\lfloor \frac{a}{2}\right\rfloor -b,1+x,\frac{x-1}{2}-b+l\right),\text{if }b=\frac{1+x}{2}\\ \left(a-1,\left\lfloor \frac{a}{2}\right\rfloor -b+l,\frac{x-1}{2}+b,\frac{x-1}{2}-b+l\right),\text{if }\frac{3+x}{2}\\ \leq b\leq l-\frac{1+a}{2}\\ \left(a-1,a-1,\frac{x-1}{2}+b,\frac{x-1}{2}-b+l\right),\text{if }l-\frac{a-1}{2}\\ \leq b\leq \frac{a-1}{2}\\ \left(b+\left\lfloor \frac{a-3}{2}\right\rfloor ,a-1,b+\frac{x-1}{2},l+\frac{x-1}{2}-b\right),\text{if }\\ \frac{a+1}{2}\leq b\leq l-\frac{x+3}{2}\\ \left(b+\left\lfloor \frac{a-3}{2}\right\rfloor ,a-1,b+\frac{x-1}{2},x\right),\text{if }l-\frac{x+1}{2}\leq b\leq l \end{array} \end{array}$$

If *a* is even, *a* = 3*p*, *p* ∈ *Z*^+^, $6\leq a\leq \left\lceil \frac{k}{2}\right\rceil$, *b* ≠ 3*q* − 1, and *q* ∈ *Z*^+^ then


$$\begin{array}{l} \;r\left(v_{a,b}|S\right)\\ =\{\begin{array}{c} \left(a-1,\left\lfloor \frac{a}{2}\right\rfloor -b+l,x,\frac{x-1}{2}-b+l\right),\text{if }1\leq b\leq \frac{x-1}{2}\\ \left(a-1,\left\lfloor \frac{a}{2}\right\rfloor -b+l,1+x,l+\frac{x-1}{2}-b\right),\text{if }b=\frac{1+x}{2}\\ \left(a-1,\left\lfloor \frac{a}{2}\right\rfloor -b+l,b+\frac{x-1}{2},\frac{x-1}{2}-b+l\right),\text{if }\frac{3+x}{2}\\ \leq b\leq l-\frac{1+a}{2}\\ \left(a-1,a-1,\frac{x-1}{2}+b,\frac{x-1}{2}-b+l\right),\text{if }l-\frac{a-1}{2}\\ \leq b\leq \frac{a-1}{2}\\ \left(b+\left\lfloor \frac{a-3}{2}\right\rfloor ,a-1,\frac{x-1}{2}+b,\frac{x-1}{2}-b+l\right),\text{if }\frac{1+a}{2}\\ \leq b\leq l-\frac{3+x}{2}\\ \left(b+\left\lfloor \frac{a-3}{2}\right\rfloor ,a-1,\frac{x-1}{2}+b,x\right),\text{if }l-\frac{1+x}{2}\leq b\leq l \end{array} \end{array}$$

If *a* is even, *a* = 3*p*, *k* ∈ *Z*^+^, $\left\lceil \frac{k}{2}\right\rceil +1\leq a\leq k$, *b* ≠ 3*q* − 1, and *q* ∈ *Z*^+^ then


$$\begin{array}{l} \;r\left(v_{a,b}|S\right)\\ =\{\begin{array}{c} \left(a-1,l+\left\lfloor \frac{a}{2}\right\rfloor -b,x,l+\frac{x}{2}-b\right),\text{if }1\leq b\leq \frac{x}{2}\\ \left(a-1,l+\left\lfloor \frac{a}{2}\right\rfloor -b,1+x,\frac{x}{2}-q+l\right),\text{if }b=\frac{x+2}{2}\\ \left(a-1,\left\lfloor \frac{a}{2}\right\rfloor -b+l,\frac{x-2}{2}+b,\frac{x}{2}-b+l\right),\text{if }\frac{x+4}{2}\\ \leq b\leq l-\frac{a-2}{2}\\ \left(a-1,a-1,\frac{x-2)}{2}+b,\frac{x}{2}-b+l\right),\text{if }l-\frac{a-4}{2}\\ \leq b\leq \frac{a}{2},b\neq 3l\\ \left(\left\lfloor \frac{a-3}{2}\right\rfloor +b,a-1,\frac{x-2}{2}+b,\frac{x}{2}-b+l\right),\text{if }\frac{2+a}{2}\\ \leq b\leq l-\frac{x+2}{2}\\ \left(b+\left\lfloor \frac{a-3}{2}\right\rfloor ,a-1,b+\frac{x-2)}{2},x\right),\text{if }l-\frac{x}{2}\leq b\leq l \end{array} \end{array}$$

**Case II:** *k* ≥ 2*l*.

If *r* ≥ 2, *rl* ≤ *k* ≤ (*r* + 1)*l*, and *k* is odd then

For *a* = *rn* + *p* where 0 ≤ *p* ≤ *l* − 1, *a* ≠ 3*p*, and *a* is odd


$$\begin{array}{l} \;r\left(v_{a,b}|S\right)\\ =\{\begin{array}{c} \left(y,y,k-a,n+\frac{k-\left(rn+p\right)}{2}-b\right),\text{if }1\leq b\leq \frac{k-\left(rl+p-2\right)}{2}\\ \left(y,y,\frac{k-\left(rl+p+2\right)}{2}+b,l+\frac{k-\left(rl+p\right)}{2}-b\right),\text{if }\frac{k-\left(rl+p-4\right)}{2}\\ \leq b\leq l-\frac{k-\left(rl+p-2\right)}{2}\\ \left(y,y,\frac{k-\left(rl+p+2\right)}{2}+b,k-a\right),\text{if }l-\frac{k-\left(rl+p\right)}{2}\leq b\leq l \end{array} \end{array}$$

If *a* is even


$$\begin{array}{l} \;r\left(v_{a,b}|S\right)\\ =\{\begin{array}{c} \left(y,y,x,\frac{k-y}{2}-b+l\right),\text{if }1\leq b\leq \frac{k-\left(rl+p-3\right)}{2}\\ \left(y,y,\frac{k-\left(rl+p+3\right)}{2}+b,\frac{k-y}{2}-b+l\right),\text{if }\frac{k-\left(rl+p-5\right)}{2}\\ \leq b\leq l-\frac{k-y}{2}\\ \left(y,y,b+\frac{k-\left(rl+p+3\right)}{2},x\right),\text{if }l-\frac{k-\left(rl+p+1\right)}{2}\leq b\leq l \end{array} \end{array}$$

If *a* = 3*p*, *a* is odd, *b* ≠ 3*q*, and *q* ∈ *Z*^+^


$$\begin{array}{l} \;r\left(v_{a,b}|S\right)\\ =\{\begin{array}{c} \left(y,y,x,\frac{k-\left(rl+p\right)}{2}-b+l\right),\text{if }1\leq b\leq \frac{k-\left(rl+p-2\right)}{2}\\ \left(y,y,b+\frac{k-\left(rl+p+2\right)}{2},\frac{k-\left(rl+p\right)}{2}-b+l\right),\text{if }\frac{k-\left(rl+p-4\right)}{2}\\ \leq b\leq l-\frac{k-\left(rl+p-2\right)}{2}\\ \left(y,y,\frac{k-\left(rl+p+2\right)}{2}+b,x\right),\text{if }l-\frac{k-\left(rl+p\right)}{2}\leq b\leq l \end{array} \end{array}$$

If *a* = 3*k*, *a* is even, *b* ≠ 3*q* − 1, and *q* ∈ *Z*^+^


$$\begin{array}{l} \;r\left(v_{a,b}|S\right)\\ =\{\begin{array}{c} \left(y,y,k-a,\frac{k-y}{2}+l-a\right),\text{if }1\leq b\leq \frac{k-\left(p+rl-3\right)}{2}\\ \left(y,y,b+\frac{k-\left(3+rl+p\right)}{2},l+\frac{k-y}{2}-b\right),\text{if }\frac{k-\left(rl+p-5\right)}{2}\\ \leq b\leq l-\frac{k-y}{2}\\ \left(y,y,\frac{k-\left(3+rl+p\right)}{2}+b,k-a\right),\text{if }l-\frac{k-\left(rl+p+1\right)}{2}\leq b\leq l \end{array} \end{array}$$

If *k* is even, *r* ≥ 2, and *rl* ≤ *k* ≤ (*r* + 1)*l* then

For *a* = *rl* + *p* where 0 ≤ *p* ≤ *l* − 1

If *a* is odd, *a* ≠ 3*p*


$$\begin{array}{l} \;r\left(v_{a,b}|S\right)\\ =\{\begin{array}{c} \left(y,x,l+\frac{k-1-rl-p)}{2}-b\right),\text{if }1\leq b\leq \frac{k-y}{2}\\ \left(y,b+\frac{k-rl-p-1)}{2},l+\frac{k-rl-p-1}{2}-b\right),\text{if }\frac{k-rl-p+3}{2}\\ \leq b\leq l-\frac{k-rl-p+3}{2}\\ \left(y,b+\frac{k-1-rl-p)}{2},x\right),\text{if }l-\frac{k-y}{2}\leq b\leq l \end{array} \end{array}$$

If *a* is even, *a* ≠ 3*p*


$$\begin{array}{l} \;r\left(v_{a,b}|S\right)\\ =\{\begin{array}{c} \left(y,y,x,l+\frac{k-\left(rl+p\right)}{2}-b\right),\text{if }1\leq b\leq \frac{k-\left(rl+p-2\right)}{2}\\ \left(y,y,b+\frac{k-\left(rl+p+2\right)}{2},l+\frac{k-\left(rl+p\right)}{2}-b\right),\text{if }\frac{k-\left(rl+p-4\right)}{2}\\ \leq b\leq l-\frac{k-\left(rl+p-2\right)}{2}\\ \left(y,y,b+\frac{k-\left(rl+p+2\right)}{2},x\right),\text{if }l-\frac{k-\left(rl+p\right)}{2}\leq b\leq l \end{array} \end{array}$$

If *a* = 3*p*, *a* is odd, *b* ≠ 3*q*, and *q* ∈ *Z*^+^


$$\begin{array}{l} \;r\left(v_{a,b}|S\right)\\ =\{\begin{array}{c} \left(y,y,x,l+\frac{k-\left(rl+p+1\right)}{2}-b\right),\text{if }1\leq b\leq \frac{k-\left(rl+p-1\right)}{2}\\ \left(y,y,b+\frac{k-1-rl-p}{2},\frac{k-1-rl-p}{2}-b+l\right),\text{if }\frac{k-\left(rl+p-3\right)}{2}\\ \leq b\leq l-\frac{k-\left(rl+p-3\right)}{2}\\ \left(y,y,\frac{k-1-rl-p}{2}+b,x\right),\text{if }l-\frac{k-\left(rl+k-1\right)}{2}\leq b\leq l \end{array} \end{array}$$

If *a* = 3*p*, *a* is even, *b* ≠ 3*q* − 1, and *q* ∈ *Z*^+^


$$\begin{array}{l} \;r\left(v_{a,b}|S\right)\\ =\{\begin{array}{c} \left(y,y,-b+k,\frac{k-\left(rl+p\right)}{2}-b+l\right),\text{if }1\leq b\leq \frac{k-\left(rl+p-2\right)}{2}\\ \left(y,y,\frac{k-2-rl-p}{2}+b,\frac{k-\left(rl+p\right)}{2}-b+l\right),\text{if }\frac{k-\left(rl+p-4\right)}{2}\\ \leq b\leq l-\frac{k-\left(rl+p-2\right)}{2}\\ \left(y,y,\frac{k-\left(rl+p+2\right)}{2}+b,x\right),\text{if }l-\frac{k-\left(rl+p\right)}{2}\leq q\leq l \end{array} \end{array}$$

These representations are distinct in at least two coordinates. So *F* is an FTRS for α_*k,l*_. Therefore $\beta^{\prime }\left(\alpha_{k,l}\right)\leq 4$. For lower bound on $\beta^{\prime }\left(\alpha_{k,l}\right)$ we discuss the following cases:

**Case I**: *k* ≥ *l*.

Since β(α_*k,l*_) = 3 [19], so $\beta^{\prime }\left(\alpha_{k,l}\right)\;>3$. Hence $\beta^{\prime }\left(\alpha_{k,l}\right)=4$ in this case.

**Case II**: *k* < *l*.

Since βα_*k,l*_ = 2 if *k* < *l*, so $\beta^{\prime }\left(\alpha_{k,l}\right)\;>2$ in this case. We claim that any set having three vertices is not an FTRS for α_*k,l*_. Let *F* = {*v*_*i,j*_, *v*_*a,b*_, *v*_*r,s*_} where *i* < *a* < *r* and *j* < *b* < *s* be an FTRS for α_*k,l*_. Then *F*_1_ = *F*\{*v*} is an R.S. for each *v* ∈ *F*. We discuss the following possibilities.

**Possibility 1**: When all vertices in *S* lie on the same row.

(i) If all vertices in *F* lie in the first row, then *i* = *a* = *r* = 1. Let *F*_1_ = {*v*_1,*j*_, *v*_1,*b*_} then *r*(*v*_1,*b*+1_|*F*_1_) = *r*(*v*_2,*b*+1_|*F*_1_), a contradiction.

(ii) If all vertices in *F* lie in *p* − *th* row and 1 < *p* ≠ 3*l, l* ∈ *Z*^+^. Let *F*_1_ = {*v*_*p,j*_, *v*_*p,b*_} then either *r*(*v*_*p,b*+1_|*F*_1_) = *r*(*v*_*p*−1,*b*+1_|*F*_1_) or *r*(*v*_*p,b*+1_|*F*_1_) = *r*(*v*_*p*+1,*b*+1_|*F*_1_), a contradiction.

(iii) If *i* = *a* = *r* = 3*q, l* ∈ *Z*^+^. Let *F*_1_ = {*v*_3*q, j*_, *v*_3*q, b*_} then either *r*(*v*_3*q* − 1, *b*+1_|*F*_1_) = *r*(*v*_3*q* + 1, *b*+1_|*F*_1_) or *r*(*v*_3*q* − 1, *b*+1_|*F*_1_) = *r*(*v*_3*q, b*+1_|*F*_1_), a contradiction.

**Possibility 2**: When two of vertices in *F* lie on the same row.

WLOG let *i* = *a* = *p* and *r* ≠ *l*. Let *F*_1_ = {*v*_*p,j*_, *v*_*p,b*_} then either *r*(*v*_*p,b*+1_|*F*_1_) = *r*(*v*_*p*−1,*b*+1_|*F*_1_) or *r*(*v*_*p,b*+1_|*F*_1_) = *r*(*v*_*p*+1,*b*+1_|*F*_1_), a contradiction.

**Possibility 3**: If all the three vertices in *F* lie on three different rows

(i) Two vertices in *F* lie on same column, let *j* = *b*. If *j* = *b* = 1 and *F*_1_ = {*v*_1,1_, *v*_*a*,1_} then *r*(*v*_2,1_|*F*_1_) = *r*(*v*_2,2_|*F*_1_), a contradiction.

(ii) If *F*_1_ = {*v*_*i*,1_, *v*_*j*,1_}, 1 ≤ *i* < *j* < *k* then either *r*(*v*_*j*,3_|*F*_1_) = *r*(*v*_*j*+1,3_|*F*_1_) or *r*(*v*_*j*,3_|*F*_1_) = *r*(*v*_*j*+1,2_|*F*_1_), a contradiction.

(iii) If *F*_1_ = {*v*_*i,b*_, *v*_*j,b*_}, 1 ≤ *i* < *j* < *k*, and 1 < *b* < *l* then either *r*(*v*_*j*+1,*b*_|*F*_1_) = *r*(*v*_*j*+1,*b*+1_|*F*_1_) or *r*(*v*_*j*+1,*b*_|*F*_1_) = *r*(*v*_*j*+1,*b*−1_|*F*_1_), a contradiction.

(iv) If all vertices in *F* lie on different columns. Let *F*_1_ = {*v*_*i,j*_, *v*_*a,b*_}, *i* < *a* then

(I) If *j* < *b* then either *r*(*v*_*a,b*+1_|*F*_1_) = *r*(*v*_*a* + 1, *b*+1_|*F*_1_) or *r*(*v*_*a,b*+1_|*F*_1_) = *r*(*v*_*a* + 1, *b*_|*F*_1_), a contradiction.

(II) If *j* > *b* then either *r*(*v*_*a*−1,*b*_|*F*_1_) = *r*(*v*_*a,b*+1_|*F*_1_) or *r*(*v*_*a*−1,*b*+1_|*F*_1_) = *r*(*v*_*a,b*+1_|*F*_1_), a contradiction.

Thus *F* is not an FTRS for α_*k,l*_. So $\beta^{\prime }\left(\alpha_{k,l}\right)\;>3$ in this case. Hence $\beta^{\prime }\left(\alpha_{k,l}\right)\;=4$.

## 3. Conclusion

In this article, we computed the fault-tolerant metric dimension of triangular and alpha boron nanotubes. In both cases, we proved that this dimension is 4. Hence, these tubes are families of a constant fault-tolerant metric dimension. These facts can be used in the networking of nano-devices using these tubes and nano-engineering.

## Author contributions

Formal analysis was done by MM. Investigation and draft are done by ZH. Both authors contributed to the article and approved the submitted version.
